# Revision of the genus *Endochilus* Weise (Coleoptera: Coccinellidae: Chilocorini)

**DOI:** 10.1093/jis/14.1.71

**Published:** 2014-01-01

**Authors:** Piotr Łączyński, Wioletta Tomaszewska

**Affiliations:** 1 Ziemiańska 35, 05-825 Grodzisk Mazowiecki, Poland; 2 Museum and Institute of Zoology, Polish Academy of Sciences; Wilcza 64, 00-679 Warszawa, Poland

**Keywords:** Africa, Cucujoidea, lady beetles, taxonomy

## Abstract

The members of the endemic African genus *Endochilus*Weise, 1898 (Coleoptera: Coccinellidae: Chilocorini) are redescribed, diagnosed, and illustrated. Lectotypes are designated for *Endochilus compater* Weise, *Endochilus minor* Weise, *Endochilus plagiatus* Sicard, *Endochilus rubicundus* Weise, and *Endochilus styx* Sicard. One new species is described: *Endochilus abdominalis***sp. nov.** Notes on the genus and nomenclatural history for each species are provided. A key for iden- tification of all species is presented. Adult characters concerning similarities of *Endochilus* to other genera of African Chilocorini are discussed.

## Introduction

*Endochilus* (Coleoptera: Coccinellidae: Chilocorini) was established by Weise (1898) for three new species from Central Africa, *Endochilus cavifrons, Endochilus minor* , and *Endochilus rubicundus* , distinguished by dark and explanate elytral margins covered with setae. Weise placed *Endochilus* in the subfamily Chilocorinae, which then included hemisphaerical beetles, usually glabrous and irregularly punctate with very short antennae, with clypeus expanded laterally and dividing the eyes, and elytral epipleura more or less foveolate.

In more modern classifications, *Endochilus* has been placed within the tribe Chilocorini Mulsant, 1846 in the subfamily Chilocorinae ( Sasaji 1968 ), which was often regarded as the “most primitive” lineage of Coccinellidae ( Sasaji 1971 ; Kovář 1996 ). According to Ślipiński (2007) , who proposed only two subfamilies for the family Coccinellidae, Microweiseinae and Coccinellinae, *Endochilus* belongs in the tribe Chilocorini of the subfamily Coccinellinae. This classification was subsequently supported by molecular and combined analyses by Giorgi et al. (2009) and Seago et al. (2011) , and was followed and implemented by Ślipiński and Tomaszewska (2010) in a comprehensive synopsis of the family Coccinellidae.

Species belonging to Chilocorini are characterized by distinctly hemispherical body, expanded clypeus, and short appendages received in repose in various fossae on ventral surfaces of the body ( Chapin 1965 ; Kovař 1995 ).

The genus *Endochilus* includes large or medi- um-sized Chilocorini possessing a labrum hidden under the clypeus and the margins of the elytra and pronotum covered with setae. Species of *Endochilus* are known only from the Afrotropical ecozone.

Previous works on *Endochilus* has been largely limited to single species descriptions, mainly by late 19th and early 20th century workers. Korschefsky’s world catalogue of Coccinellidae ( Korschefsky 1932 ) listed eight species of *Endochilus* . Apart from Chapin (1965) , who reviewed the genera of Chilocorini based on a study mostly of single (type) species for each genus and redescribed *Endochilus* based on *E. plagiatus* Sicard, there have been no recent publications including *Endochilus* .

During preparation of the present review, a study of the type specimen of *Endochilus meridionalis*Sicard, 1929 , led us to discover several features unusual for *Endochilus* . Three-segmented tarsi, 11-segmented antennae, and mandible bidentate apically found in *E. meridionalis* are unique for the tribe Chilocorini. Therefore this species was recently removed from *Endochilus* and a new genus of Chilocorini, *Chapinaria* Łączyński *et* Tomaszewska, 2012 was established and described ( Łączyński and Tomaszewska 2012 ).

In the present paper, 11 species of *Endochilus* are recognized as valid along with *E. abdominalis***sp. nov.** , described here, all distributed along the Gulf of Guinea and throughout the Valley of Congo.

The present paper provides the first comprehensive treatment of the entire genus with a key to all species and detailed morphological characters allowing their identification.

## Materials and Methods

This study was based on examination of the type and non-type specimens, borrowed from the following museums:

MIZ – Muzeum i Instytut Zoologii PAN, Warszawa, Poland

MNB – Museum für Naturkunde, Berlin, Germany

MNHN – Museum National d’Histoire Naturelle, Paris, France

NHM – The Natural History Museum, London, England

RMCA – Royal Museum for Central Africa, Tervuren, Belgium

ZSM – Zoologische Staatssammlung München, Germany

Measurements were made using an ocular micrometer attached to an Olympus SZH 10 (( www.olympus-global.com ) dissecting microscope and are defined as follows: (TL) total length, from apical margin of clypeus to apex of elytra; (PL) pronotal length, from the middle of anterior margin to base of pronotum; (PW) pronotal width, at widest part; (EL) elytral length, along suture including scutellum; (EW) elytral width, across both elytra at widest part; (PSL) prosternal length, prosternum length anterior to procoxa; and (PPW) prosternal process width, at widest point of prosternal process.

Male and female genitalia were dissected, cleared in a 10% solution of KOH, and placed in glycerine on slides for further study. Structural illustrations were made from slide preparations using a camera lucida attached to a Leica dissecting microscope.

SEM micrographs were made using a Hitachi S-3400N machine (( www.hitachi.com ), and habitus images were captured using a digital camera mounted on a Leica microscope (( www.leicamicrosystems.com ) and subsequently enhanced using Auto-Montage software (Syncroscopy, ( www.syncroscopy.com ) in the Electron Microscopy Laboratory of the MIZ.

Terminology used for adult morphology follows Ślipiński (2007) and Ślipiński and Tomaszewska (2010) .

The label data under material examined are given “in verbatim”.

### Nomenclature

This publication and the nomenclature it contains have been registered in Zoobank. The LSID number is:

urn:lsid:zoobank.org:pub:F3B4BD0D-65AB-449F-AAD0-1DE76354F74F

It can be found online by inserting the LSID number after ( www.zoobank.org/

## Results

### Genus and species descriptions

**Genus** *Endochilus* Weise, 1898

*Endochilus* Weise, 1898 : 119. Type species, by subsequent designation of Korschefsky, 1932 : *Endochilus cavifrons*Weise, 1898 .

**Diagnosis** . Within the tribe Chilocorini, *Endochilus* can be easily distinguished from other taxa by the following combination of characters: labrum hidden under clypeus; clypeus expanded laterally deeply into eyes and almost dividing each eye into two parts; antennae 8-segmented, short; distinct and deep antennal grooves for reception of 3rd and 4th antennomeres; pronotum wholly or partly covered with setae; margins of elytra broadly explanate, but not abruptly reflexed, and covered with setae.

**Description.** Length 2.89–6.10 mm. Body rounded and convex; pronotal margin moderately to very broad; elytral margins moderately to widely explanate; both entirely visible from above. Pronotum black, blackish, bright red to dark red; elytra black, bright red to dark red. Punctures on pronotum and elytra about as large as eye facets; these on pronotum moderately deep, on elytra shallower; surfaces between elytral and pronotal punctures polished and shiny; dorsum apparently glabrous except pronotal and elytral margins, which are covered with setae (in some taxa entire pronotum setose). Ventral surface reddish, dark reddish to brown or black.

Head subquadrate; ventral antennal grooves distinct, deep and short, receive 3rd and 4th antennomeres ( Figures 119 , 138 , 153 ). Eye finely faceted. Clypeus expanded laterally deeply into eyes, almost divides each eye into two parts ( Figures 117 , 156 , 162 ). Labrum hidden under clypeus ( Figures 117 , 151 , 162 ). Antenna 8-segmented (Figures 119 , 138 , 153 ), sparsely covered with long setae; scape and pedicel entirely hidden under clypeal shelf; scape simple, obconical in shape; pedicel bar- rel-shaped, as long as scape, tapering to apex; antennomere 3 obconical, antennomeres 4 and 5 same length, antennomere 6 as long as 4 and 5 together, antennomere 7 between 1.15–2.00 times as long as terminal. Mentum trapezoidal, covered with sparse, long setae. Prementum between labial palps narrow with few long setae visible. Labial palp 3- segmented; labial terminal palpomere strongly tapering apically, narrowed, truncate at apex, at base as wide as penultimate palpomere at apex. Maxilla with transverse cardo and sub- triangular stipes; maxillary palp 4-segmented; terminal palpomere with apex obliquely truncate. Mandible with strong, acute apical tooth.

Prothorax strongly descending anteriorly; anterior margin deeply emarginate with anterior angles and lateral margins rounded ( Figures 106 , 124 , 133 ); pronotal base usually without bordering line; hypomeron with more or less distinct fovea; prosternal process subtruncate or rounded apically.

Meso-metaventral junction almost straight and very broad. Metaventrite with nearly complete discrimen; postcoxal lines separated medially, weakly descending posteriorly, at distal end each slightly recurved anteriorly. Metepimer- on indistinct. Scutellum triangular, very small, without setae. Elytral epipleuron broad between 9–11 times metanepisternum width, with maximum width at metaventrite level narrowing posteriorly, complete to apex with shallow or distinct foveae. Wings welldeveloped.

Legs moderately stout; mid and hind tibiae simple ( Figures 110 , 115 , 134 ); tarsal claws simple, slightly swollen at base ( Figures 127 , 131 ); empodium without setae.

Abdomen with 5 or 6 ventrites in male, 5 in female; intercoxal process smooth or distinctly punctate, punctures about as large as eye facets; postcoxal lines usually separated medially, curved posteriorly, closely paralleling posterior margin, incomplete laterally; ventrite 1 along midline between 1.85–2.90 times longer than ventrite 2; ventrite 5 with posterior margin truncate or emarginate in male, rounded in female. Abdominal segment VIII with posterior margin of sternite deeply emarginate medially in male, rounded or somewhat narrowly truncate or sometimes weakly emarginate in female. Male genital segment usually with long apophysis, at base distinctly or weakly swollen, narrow and simple at apex.

Male genitalia with tegminal basal piece with distinct strut ( Figures 15 , 24 , 91 ); parameres long and thin, densely setose along at least distal half of their length ( Figures 24 , 33 , 91 ); penis guide lanceolate ( Figures 24 , 57 , 65 ), slightly longer, shorter or as long as parameres; penis slender of uniform diameter throughout most of its length with large basal capsule ( Figures 37 , 77 , 104 ).

Female genitalia with ovipositor well sclero- tised, coxites elongate without styli, only with long setae visible ( Figures 17 , 26 , 59 ); proper infundibulum absent but appendix of bursa copulatrix present in form of membranous protuberance at outlet of sperm duct ( Figures 17 , 59 , 102 ); spermatheca bean-shaped ( Figures 18 , 27 , 36 ) without distinct ramus and nodulus; spermathecal gland (when present) subcircular; sperm duct short of two different diameters ( Figure 59 ).

**Figures 54-62.**
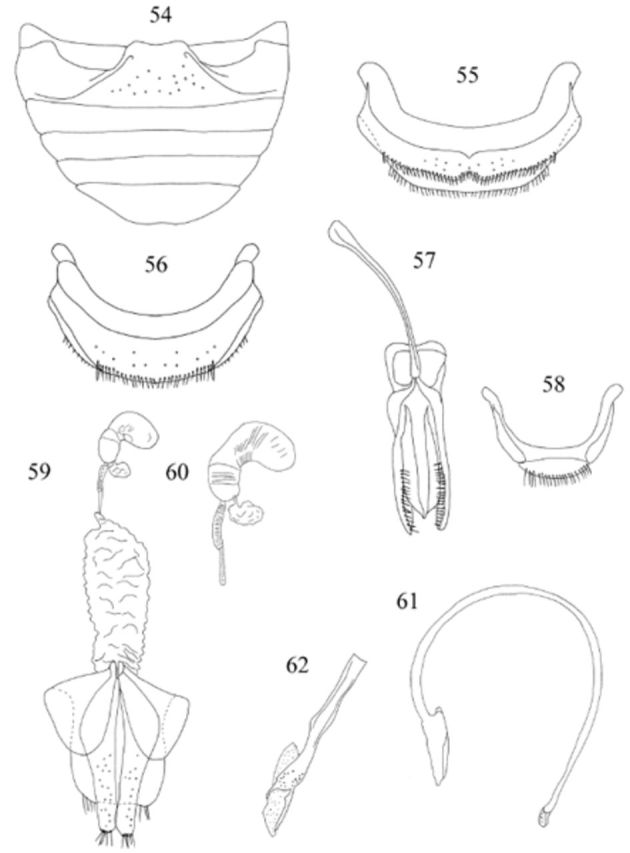
*Endochilus minor* Weise. 54: abdomen, male, ventral; 55: abdominal segment VIII, male, ventral; 56: abdominal segment VIII, female, ventral; 57: tegmen, inner view; 58: male genital segment, ventral; 59: female genitalia; 60: spermatheca; 61: penis; 62: apex of penis. High quality figures are available online.

**Distribution.** *Endochilus* is entirely comprised of African species, with its greatest diversity in the West and Central Africa.

**Species treatments**

*Endochilus abdominalis* **sp. nov.**

( Figures 1 , 12–20 , 106–110 )

**Diagnosis** . Abdominal postcoxal lines separated medially by distance about 0.3 width of intercoxal process and apex of penis with subacute projection placed on inner margin distinguish this species from all its congeners.

**Description** . Length 4.3–4.5 mm; TL/EW = 0.97–1.02; PL/PW = 0.38–0.40; EL/EW = 0.76–0.80; PSL/PPW = 0.62.

**Figures 1-11.**
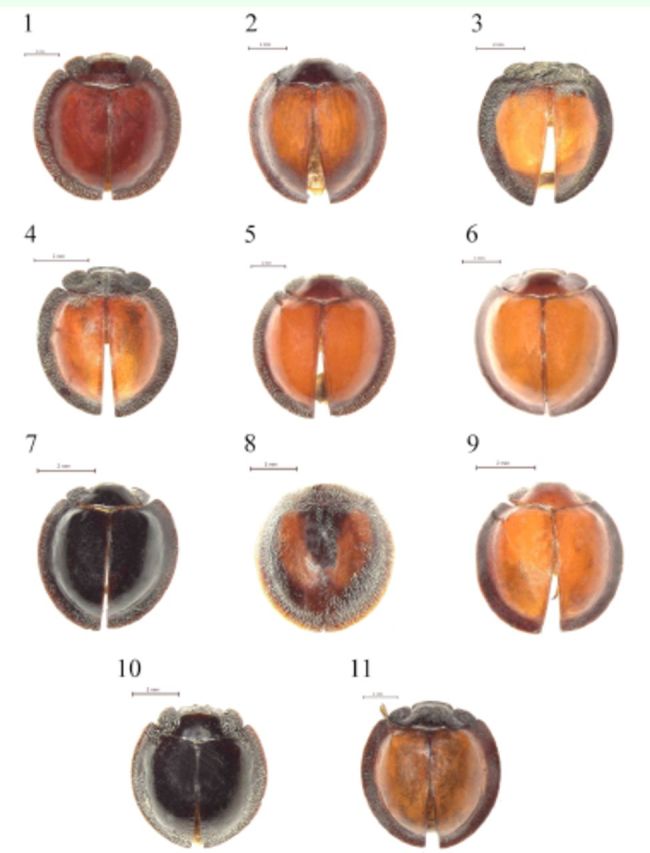
*Endochilus* spp. Habitus, dorsal. 1: *E. abdominalis***sp. nov.** ; 2: *E. brunneocinctus* Sicard; 3: *E. cavifrons* Weise; 4: *E. compater* Weise; 5: *E. epipleuralis* Mader; 6: *E. minor* Weise; 7: *E. niger* Fürsch; 8: *E. plagiatus* Sicard; 9: *E. rubicundus* Weise; 10: *E. styx* Sicard; 11: *E. weisei* Mader. Scale bar = 1 mm ( Figures 1 , 2 , 5 , 6 , 8 , 10 , 11 ); scale bar = 2 mm ( Figures 3 , 4 , 7 , 9 ). High quality figures are available online.

Body ( Figure 1 ) with pronotal margins very broad; elytral margins widely explanate. Head and pronotum dark red; labrum, ventral mouthparts and antennae brownish. Scutellum and elytra predominantly very intense dark red. Punctures on pronotum 2.0–2.5 diameters apart; punctures on elytra 2.5–3.0 diameters apart; dorsum glabrous except pronotal angles and elytral margins which are covered with setae. Ventral surface dark reddish to brownish.

**Figures 106-115.**
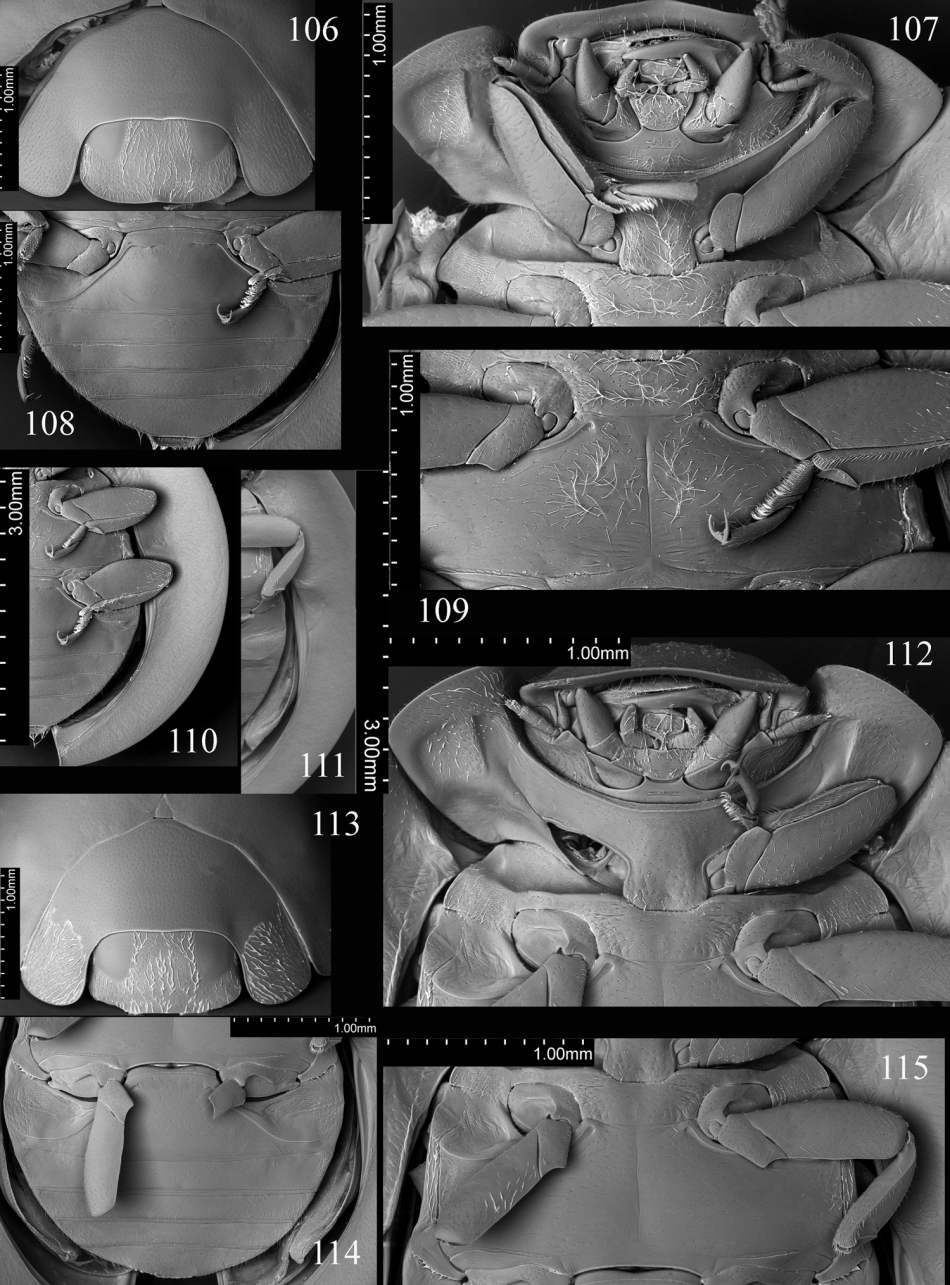
*Endochilus abdominalis* , **sp. nov.** 106: head, prothorax and base of elytra, anterodorsal; 107: head, pro- and mesothorax, ventral; 108: abdomen, male, ventral; 109: meso- and metathorax, ventral; 110: legs and elytral epipleuron. *E. brunneocinctus* Sicard. 111: legs and elytral epipleuron; 112: head, pro- and mesothorax, ventral; 113: head, prothorax and base of elytra, anterodorsal; 114: abdomen, female, ventral; 115: meso- and metathorax, ventral. High quality figures are available online.

Head flat medially, punctate, covered with rather dense and moderately long setae. Clypeus ( Figure 106 ) length anterior to eyes about 0.23 times head width, weakly arcuate anteriorly, weakly reflexed in the middle of anterior margin. Eyes large; interocular distance nearly 0.45 times head width; medial margins of eyes slightly rounded, divergent anteriorly. Maxillary terminal palpomere ( Figure 107 ) about 1.5 times longer than wide, lateral margin about 3.75 times as long as medial, subparallel along basal 1/4 of its length, strongly tapering apically. Antenna as in Figure 107 with penultimate antennomere about 1.15 times as long as terminal segment.

Prothorax about 0.8 times basal width of elytra; pronotal hypomeron with distinct fovea; prosternum smooth; prosternal process ( Fig ure 107 ) covered with sparse long setae. Mesoventral process ( Figure 109 ) about 1.7 times mesocoxal longitudinal diameter, covered with sparse long setae. Metaventrite covered with sparse long setae. Elytral epipleuron ( Figure 110 ) about 11 times metanepisternum width, with shallow foveae ( Figure 111 ).

Abdomen ( Figures 12 , 108 ) with 5 ventrites in both sexes; postcoxal lines of first ventrite ( Figure 108 ) separated medially by distance equal 0.3 width of intercoxal process; ventrite 1 along midline about 2.75 times longer than ventrite 2; ventrite 5 with posterior margin truncate in male, rounded in female. Abdominal segment VIII ( Figures 13 , 14 ) with posterior margin of sternite emarginate medially in male, subtruncate in female. Male genital segment ( Figure 16 ) with apophysis at base distinctly swollen.

Male genitalia as in Figures 15 , 19 , 20 . Penis guide slightly longer than parameres; apex of penis ( Figure 20 ) with subacute projection along inner margin. Female genitalia as in Figures 17 , 18 . Spermatheca with moderately large spermathecal gland.

**>Figures 12-20.**
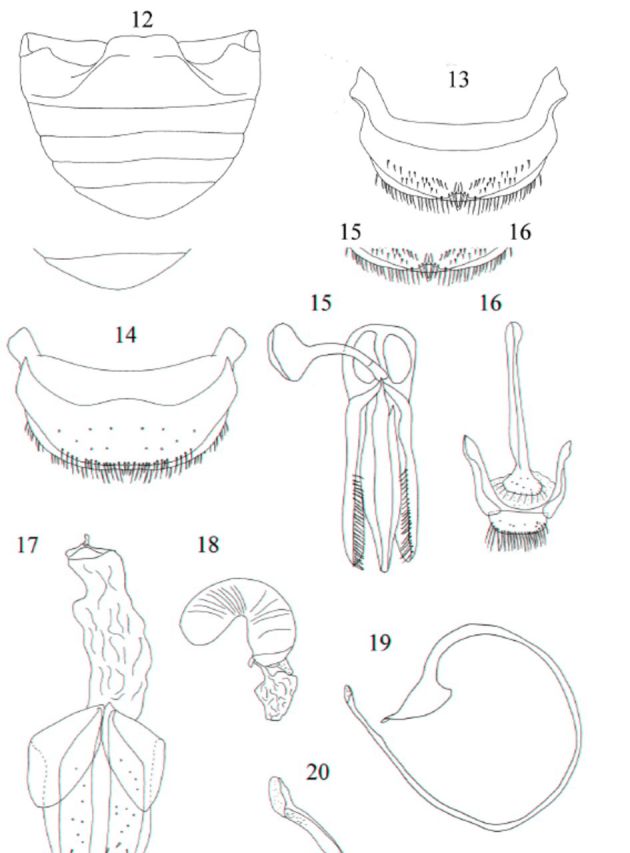
*Endochilus abdominalis* , **sp. nov.** 12: abdomen, male, ventral; 13: abdominal segment VIII, male, ventral; 14: abdominal segment VIII, female, ventral; 15: tegmen, inner; 16: male genital segment, ventral; 17: female genitalia; 18: spermatheca; 19: penis; 20: apex of penis. High quality figures are available online.

### Material examined

**Type material.** *Holotype* (male): “N.W. Kamerun, Moliwe b. Victoria, 17.I. – 7.III – 08, Frfr.v.Maltzan G.” (MNB). ****Paratypes**** : “N.W. Kamerun, Moliwe b. Victoria, 17.I. – 7.III – 08, Frfr.v.Maltzan G.” (1: MIZ); “N.W. Kamerun, Moliwe b. Victoria, 17.I. – 7.III – 08, Frfr.v.Maltzan G.” (1: MNB); “Endochilus rubricundus, Spanish Guinea” (1: MNB); “Span-Guinea Nkolentangan, IX.07-V.08, G. Teβmann S.G.” (1: MNB); “Fernando Poo, Sa-Jsabel, 21.5.1900, L. Conradt S.” (1: MNB); “MUSEUM PARIS, COURS DU CONGO, Entre Leopoldville, Stanleyville, L. BURGEON 1918” (1: MNHN).

**Etymology.** The name of this species refers to the shape of the abdominal postcoxal lines, which are narrowly separated on intercoxal process, differently than in other *Endochilus* species.

**Distribution.** Cameroon, Equatorial Guinea. *Endochilus brunneocinctus* Sicard ( Figures 2 , 21–29 , 111–115 ) *Endochilus brunneocinctus*Sicard, 1930 : 73.

**Diagnosis** . Combination of weakly explanate margins, blackish pronotum, dark red coloration of elytra and apex of penis with acute projection along outer margin distinguish this species from all its congeners.

**Description** . Length 3.8–4.0 mm; TL/EW = 1.00–1.05; PL/PW = 0.30–0.34; EL/EW = 0.80–0.85; PSL/PPW = 0.52.

Body ( Figure 2 ) with pronotal margins moderately broad; elytral margins weakly explanate. Head and pronotum blackish; labrum, ventral mouthparts and antennae brownish. Scutellum dark red. Elytra predominantly dark red. Punctures on pronotum 2–3 diameters apart; punctures on elytra 3–4 diameters apart; dorsum glabrous except pronotal angles and elytral margins which are covered with short and sparse setae. Ventral surface dark red to dark brown.

Head flat medially, punctate, covered with rather dense and moderately long setae. Clypeus ( Figure 113 ) length anterior to eyes about 0.18 times head width, with straight anterior margin. Eyes large; interocular distance nearly 0.42 times head width; medial margins of eyes slightly rounded, divergent anteriorly. Maxillary terminal palpomere ( Figure 112 ) about 1.8 times longer than wide, lateral margin about 3 times as long as medial, subparallel along basal 1/3 of its length, tapering apically. Antenna as in Figure 112 , with penultimate antennomere about 1.2 times as long as terminal antennomere.

Prothorax about 0.9 times basal width of elytra; pronotal hypomeron with distinct fovea; prosternum smooth; prosternal process ( Figures 112 , 115 ). Mesoventral process ( Figure 115 ) about 1.5 times mesocoxal longitudinal diameter. Elytral epipleuron ( Figure 111 ) about 9.25 times metanepisternum width, with distinct foveae.

**Figures 21-29.**
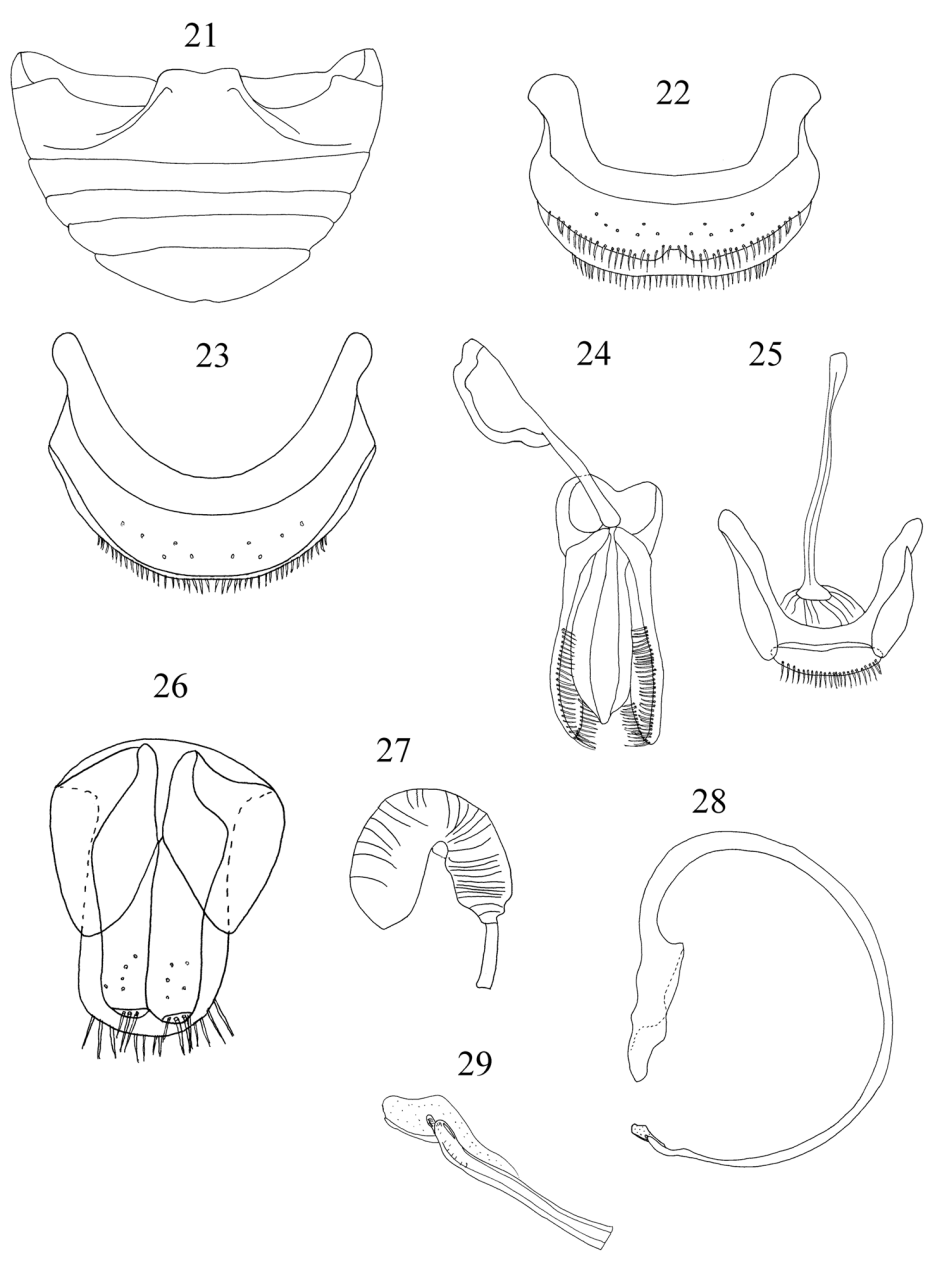
*Endochilus brunneocinctus* Sicard. 21: abdomen, male, ventral; 22: abdominal segment VIII, male, ventral; 23: abdominal segment VIII, female; 24: tegmen, inner view; 25: male genital segment, ventral; 26: female genitalia; 27: spermatheca; 28: penis; 29: apex of penis. High quality figures are available online.

Abdomen ( Figures 21 , 114 ) with 5 ventrites in both sexes; intercoxal process punctate, 3–4 diameters apart; ventrite 1 along midline about 2.9 times longer than ventrite 2; ventrite 5 with posterior margin weakly emarginate in male, rounded in female. Abdominal segment VIII ( Figures 22 , 23 ) with posterior margin of sternite emarginate medially in male, rounded in female. Male genital segment ( Figure 25 ) with apophysis at base distinctly swollen.

Male genitalia as in Figures 24 , 28 , 29 . Penis guide slightly shorter than parameres; apex of penis ( Figure 29 ) with acute projection placed outwardly.

Female genitalia as in Figures 26 , 27 . Spermathecal gland absent.

### Material examined

**Type material.** ***Holotype*** (female): “Holotypus/ Musee du Congo, Eala, I-II-1917, R. Mayne/ R. Det. A. 1607/ Endochilus brunne- cinctus Sic.:” (RMCA).

**Other material** : “Gabon, Bas-Ogoocue, Ex- collection Favarel, Endochilus brunneocintcus Sic. Sp. N., Museum Paris 1930, Coll. Sicard” (1: MNHN); “Museum Paris 1930, Coll. Sicard” (1: MNHN); “Joko Kamerun, Museum Paris 1930, Coll. Sicard” (1: MNHN); “The Sanga Carnot, Museum Paris 1930, Coll. Sicard” (1: MNHN); Congo belge Centr Kassai Edm. Tayma’s 1904, Paris 1930, Coll. Sicard, K Oberthür 1952” (1: MNHN); “Kamerun, Joh-, Albrechtshőhe, 29.VIII-13.IX.98, L. Conradt, Endochilus brunneocinctus det. Mader” (1: ZSM).

**Distribution.** Cameroon, Gabon, Democratic Republic of the Congo.

*Endochilus cavifrons* Weise

( Figures 3 , 30–38 , 116–123 )

*Endochilus cavifrons* Weise, 1898 : 120.

**Diagnosis** . This species is very similar to *E. compater* but is easily distinguished by black base of elytra, emarginate posterior margin of ventrite 5 in female, and peculiar shape of penis. Penis with row of teeth placed on outer margin before apex, and apex with stout beak bent externally distinguish this species from all its congeners.

**Description** . Length 5.9–6.1 mm; TL/EW = 0.98–1.02; PL/PW = 0.22–0.24; EL/EW = 0.90–0.95; PSL/PPW = 0.56.

Body ( Figure 3 ) with pronotal margins very broad; elytral margins widely explanate. Head and pronotum black; labrum, ventral mouthparts and antennae brownish. Scutellum black. Elytra predominantly bright red except bases, apices, and lateral margins, which are black. Punctures on pronotum 1.5–2.0 diameters apart; punctures on elytra 2–3 diameters apart; dorsum glabrous except pronotum, elytral base, and margins, which are covered with setae. Ventral surface dark red to brown.

Head flat medially, punctate, covered with dense and long setae. Clypeus ( Figures 121 , 123 ) length anterior to eyes about 0.25 times head width, weakly arcuate anteriorly. Eyes large; interocular distance nearly 0.45 times head width; medial margins of eyes slightly rounded, almost parallel. Maxillary terminal palpomere ( Figures 117 , 119 ) about 1.45 times longer than wide, lateral margin about 2.8 times as long as medial, subparallel along basal 1/3 of its length, moderately tapering apically. Antenna ( Figure 119 ) with penultimate antennomere about 2 times as long as terminal antennomere.

**Figures 116-123.**
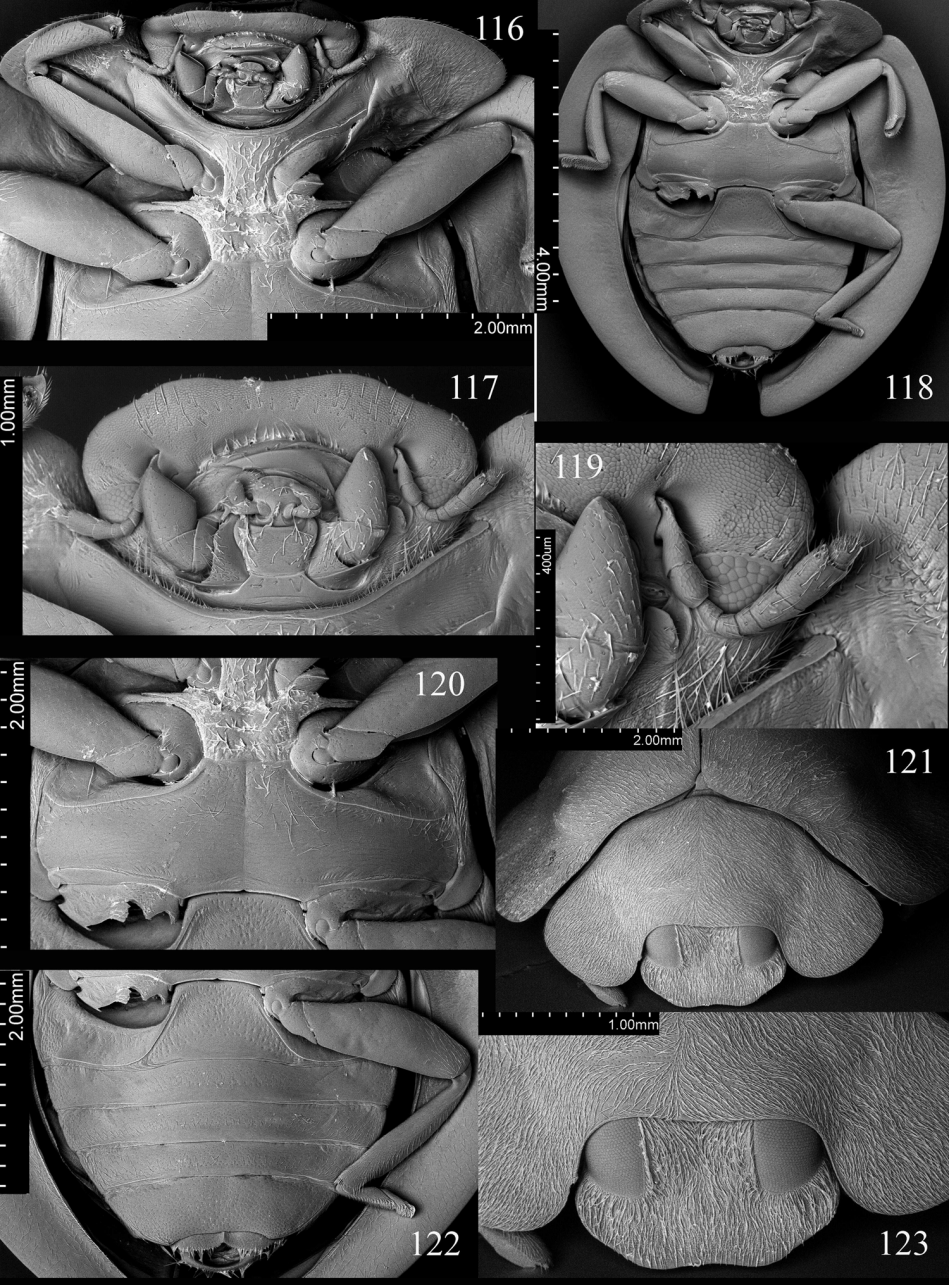
*Endochilus cavifrons* Weise. 116: head, pro- and mesothorax, ventral; 117: head, ventral; 118: body, ventral view; 119: antenna; 120: meso- and metathorax, ventral; 121: head, prothorax and base of elytra, anterodorsal; 122: abdomen, male, ventral; 123: head, anterodorsal. High quality figures are available online.

Prothorax about 0.9 times basal width of elytra; pronotal hypomeron with shallow fovea; prosternum smooth; prosternal process ( Figures 116 , 120 ) covered with sparse long setae. Mesoventral process ( Figure 116 , 120) about 0.85 times mesocoxal longitudinal diameter. Elytral epipleuron ( Figure 118 ) about 11 times metanepisternum width, with shallow foveae.

**Figures 30-38.**
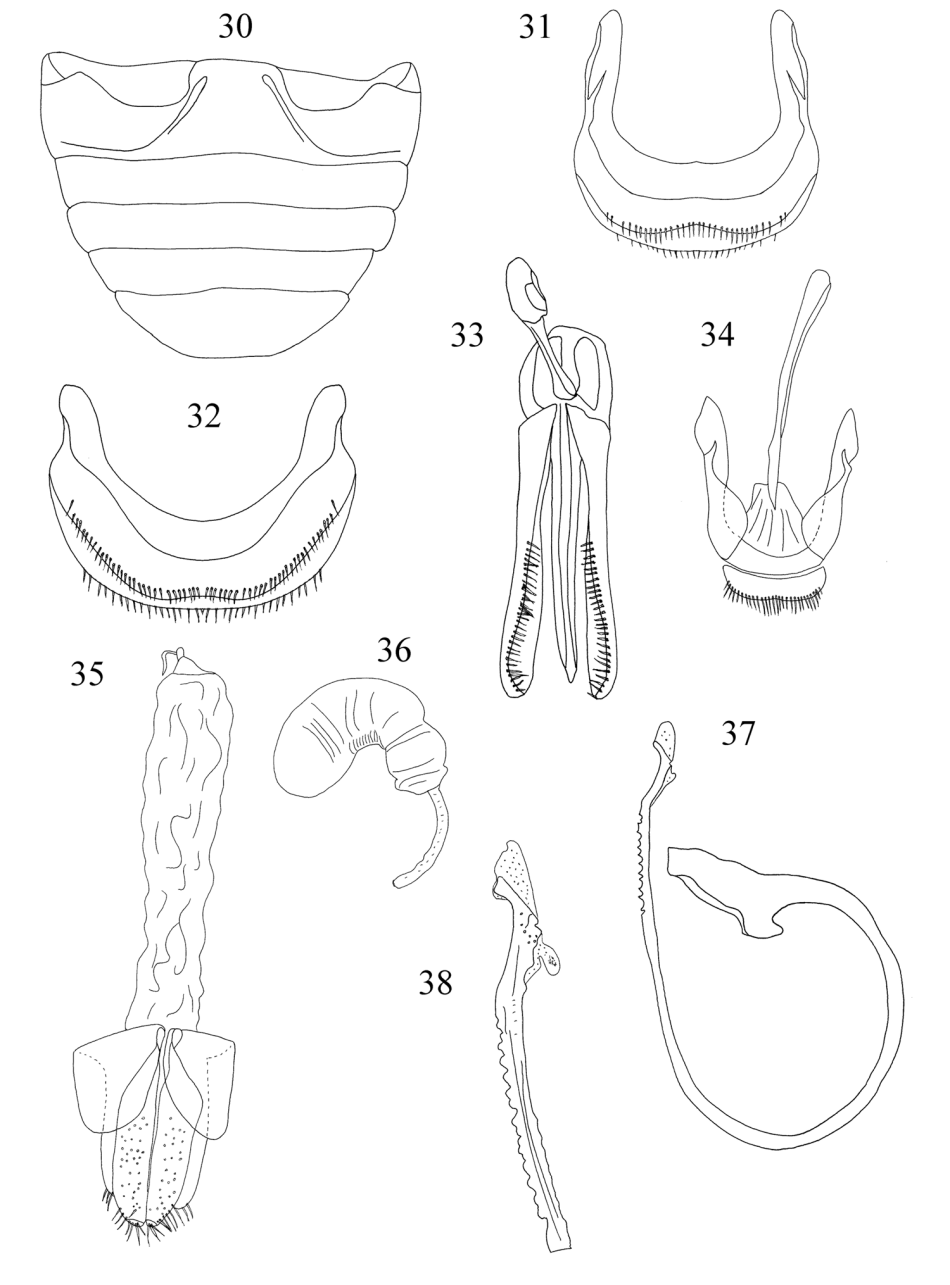
*Endochilus cavifrons* Weise. 30: abdomen, female, ventral; 31: abdominal segment VIII, male, ventral; 32: abdominal segment VIII, female, ventral; 33: tegmen, inner view; 34: male genital segment, ventral; 35: female genitalia; 36: spermatheca; 37: penis; 38: apex of penis. High quality figures are available online.

Abdomen ( Figures 30 , 118 , 122 ) with 6 ventrites in male, 5 in female; intercoxal process distinctly punctate, punctures 2–3 diameters apart; ventrite 1 along midline about 1.95 times longer than ventrite 2; ventrite 5 with posterior margin emarginate in male, narrowly truncate in female. Abdominal segment VIII with posterior margin of sternite emarginate medially in both sexes ( Figures 31 , 32 ). Male genital segment ( Figure 34 ) with long apophysis, narrow, simple at base and at apex.

Male genitalia as in Figures 33 , 37 , 38 . Penis guide as long as parameres; penis with row of teeth placed on outer margin before apex; apex of penis ( Figure 38 ) with stout beak bent externally.

Female genitalia as in Figures 35 , 36 . Spermathecal gland absent.

### Material examined

**Type material** . ****Holotype**** (male): “Kamerun, Jaunde-Stat./Endochilus cavifrons m./Holotypus, Endochilus cavifrons Weise, 1898 , labeled by MNHUB 2010.” (MNB).

**Other material** : “Endochilus cavifrons m., Kamerun, Mundame” (1: MNB); “Span. Guinea, Nkolentangan, XI.07 – V.08., G. Teβmann S.G.” (10: MNB).

**Distribution.** Cameroon.

*Endochilus compater* Weise

( Figures 4 , 39–47 , 124–129 )

*Endochilus compater* Weise, 1910 : 46.

**Diagnosis** . This species is most similar to *E. cavifrons* but can be distinguished by having red bases of elytra, rouned posterior margin of ventrite 5 in female, and penis without subapical serration. Penis with stout and sinuate apex distinguishes this species from its congeners.

**Description** . Length 5.3–5.6 mm; TL/EW = 1.03–1.05; PL/PW = 0.26–0.29; EL/EW = 0.84–0.88; PSL/PPW = 0.65.

Body ( Figure 4 ) with pronotal margins very broad; elytral margins widely explanate. Head and pronotum black; labrum, ventral mouthparts and antennae brownish. Scutellum black. Elytra predominantly bright red except margins, which are black. Punctures on pronotum 1.5–2.0 diameters apart; punctures on elytra 2–3 diameters apart; dorsum glabrous except pronotum and elytral margins, which are covered with setae. Ventral surface dark red to dark brown.

**Figures 124-135.**
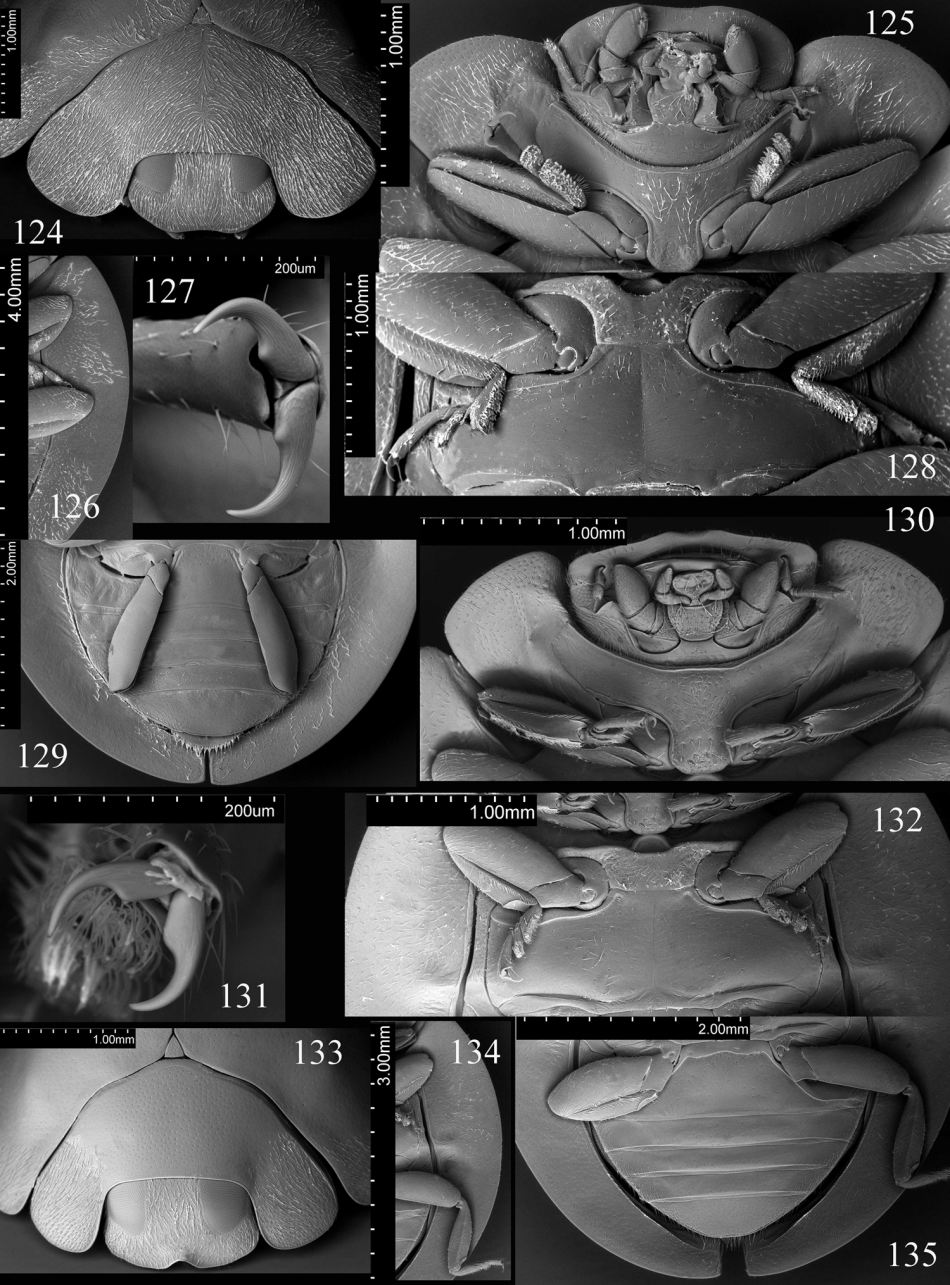
*Endochilus compater* Weise. 124: head, prothorax and base of elytra, anterodorsal; 125: head, prothorax, ventral; 126: elytral epipleuron; 127: tarsal claws; 128: meso- and metathorax, ventral; 129: abdomen, female, ventral. *E. epipleuralis* Mader. 130: head, prothorax, ventral; 131: tarsal claws; 132: meso- and metathorax, ventral; 133: head, prothorax and base of elytra, anterodorsal; 134: elytral epipleuron; 135: abdomen, male, ventral. High quality figures are available online.

Head flat medially, punctate, covered with dense and long setae. Clypeus ( Figure 124 ) length anterior to eyes about 0.25 times head width, weakly arcuate anteriorly. Eyes large; interocular distance nearly 0.4 times head width; medial margins of eyes slightly rounded, almost parallel. Maxillary terminal palpomere ( Figure 125 ) about 1.6 times longer than wide, lateral margin about 2.7 times as long as medial, subparallel along basal 1/3 of its length, moderately tapering apically. Antenna as in Figure 119 with penultimate antennomere about 2.4 times as long as terminal.

Prothorax about 0.9 times basal width of elytra; pronotal hypomeron without fovea; prosternum smooth; prosternal process ( Figure 125 ). Mesoventral process ( Figure 128 ) about 0.83 times mesocoxal longitudinal diameter. Elytral epipleuron ( Figure 126 ) about 11 times metanepisternum width, without foveae.

**Figures 39-47.**
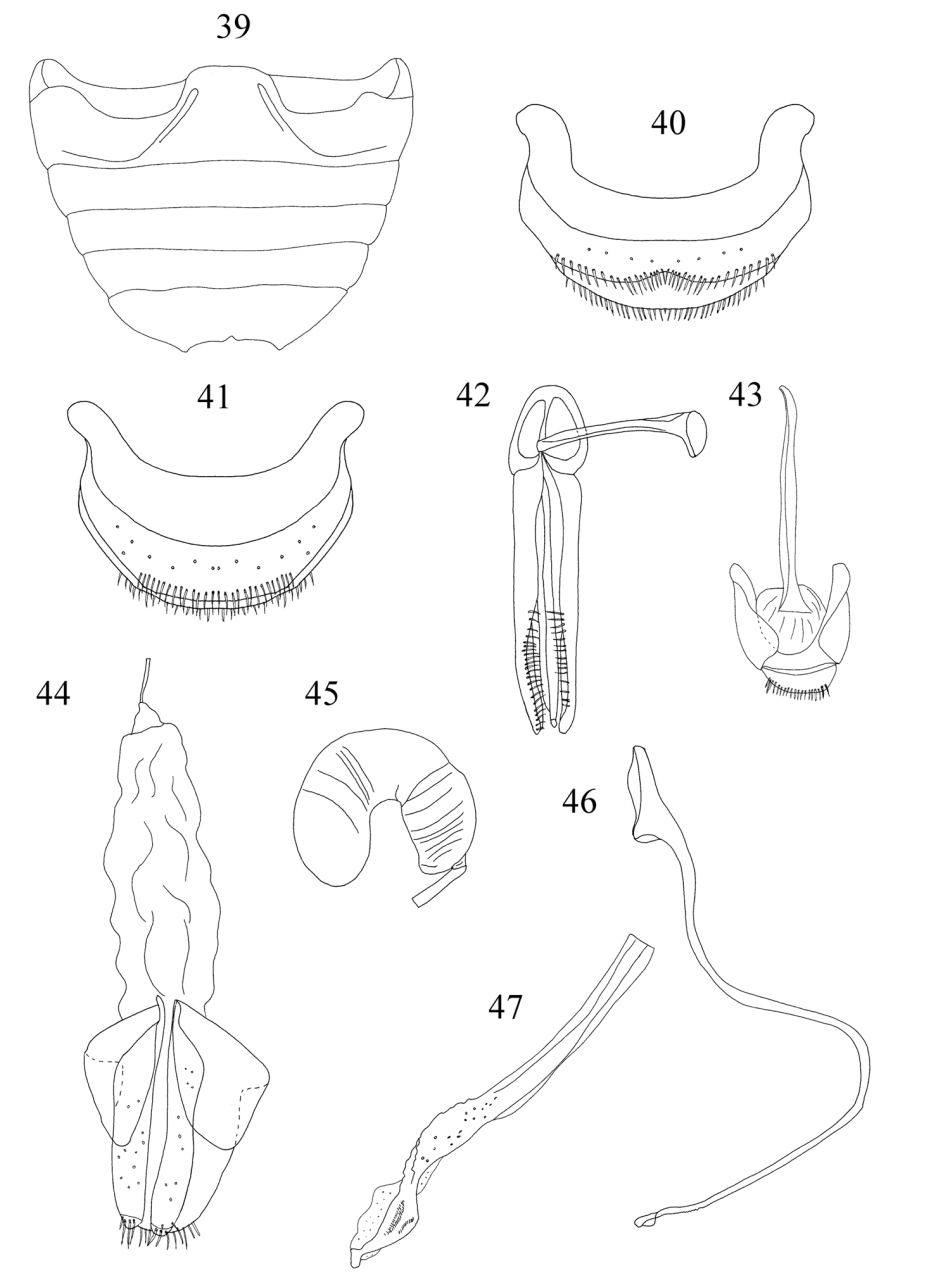
*Endochilus compater* Weise. 39: abdomen, male, ventral; 40: abdominal segment VIII, male, ventral; 41: abdominal segment VIII, female, ventral; 42: tegmen, inner view; 43: male genital segment, ventral; 44: female genitalia; 45: spermatheca; 46: penis; 47: apex of penis. High quality figures are available online.

Abdomen ( Figures 39 , 129 ) with 6 ventrites in male, 5 in female; intercoxal process distinctly punctate, punctures 2–3 diameters apart; ventrite 1 along midline about 1.85 times longer than ventrite 2; ventrite 5 with posterior margin strongly emarginate with additional medial incision in male, rounded in female; male ventrite 6 emarginate ( Figure 40 ). Abdominal segment VIII in female ( Figure 41 ) with posterior margin of sternite rounded. Male genital segment ( Figure 43 ) with apophysis swollen at base.

Male genitalia as in Figures 42 , 46 , 47 . Penis guide as long as parameres; apex of penis ( Figure 47 ) stout and sinuate.

Female genitalia as in Figures 44 , 45 . Spermathecal gland absent.

### Material examined

**Type material.** *Lectotype (here designated)* (female): “Kamerun, Mundame/Mundame dr. Schulz/SYNTYPUS, Endochilus compater Weise, 1910 , labeled by MNHUB 2010” (MNB). ****Paralectotypes**** : “Kamerun, Mundame/Mundame dr. Schulz/SYNTYPUS, Endochilus compater Weise, 1910 , labeled by MNHUB 2010” (1: MNB); “Kamerun, Mundame/Mundame (Kam.) R. Rohde/SYNTYPUS, Endochilus compater Weise, 1910 , labeled by MNHUB 2010” (1: MNB); “Kamerun, Mundame/Mundame dr. Schulz/Endochilus compater/SYNTYPUS, Endochilus compater Weise, 1910 , labeled by MNHUB 2010” (1: MNB).

**Other material** : “Fernando Poo, Sa-Jsabel, 21.5.1900, L. Conradt S.” (1: MNB); “Kamerun Barembi, Conradt/Endochilus compater Ws., det. R. Korschefsky 1939 (1: MIZ).

**Distribution.** Cameroon.

*Endochilus epipleuralis* Mader

( Figures 5 , 48–53 , 130–135 )

*Endochilus epipleuralis* Mader, 1954 : 68.

**Diagnosis** . This is the only species of *Endochilus* with abdominal postcoxal lines joined medially on intercoxal process.

**Description** . Length 4.2; TL/EW = 1.03; PL/PW = 0.30; EL/EW = 0.85; PSL/PPW = 0.70.

Body ( Figure 5 ) with pronotal margins broad; elytral margins moderately explanate. Head and pronotum dark red; labrum, ventral mouthparts and antennae brownish. Scutellum dark red. Elytra predominantly bright red except dark red margins. Punctures on pronotum 2.0–2.5 diameters apart; punctures on elytra as on pronotum but shallower; dorsum glabrous except pronotal angles and elytral margins which are covered with setae. Ventral surface dark reddish.

Head flat medially, punctate, covered with rather dense and moderately long setae. Clypeus ( Figure 133 ) length anterior to eyes almost 0.2 times head width, with deep emargination in the middle of anterior margin, weakly reflexed along anterior margin. Eyes large; interocular distance about 0.38 times head width; medial margins of eyes slightly rounded, almost parallel. Maxillary terminal palpomere ( Figure 130 ) about 1.5 times longer than wide, lateral margin about 2.15 times as long as medial, subparallel along basal 1/2 of its length, moderately tapering apically. Antenna as in Figure 130 with penultimate antennomere about 1.52 times as long as terminal antennomere.

Prothorax about 0.9 times basal width of elytra; pronotal base bordered; pronotal hypomeron without fovea; prosternum smooth; prosternal process ( Figure 130 ) covered with sparse long setae. Mesoventral process ( Figure 132 ) about 1.3 times mesocoxal longitudinal diameter, covered with sparse long setae. Metaventrite covered with sparse long setae. Elytral epipleuron ( Figure 134 ) about 9.6 times metanepisternum width, with distinct foveae.

**Figures 48-53.**
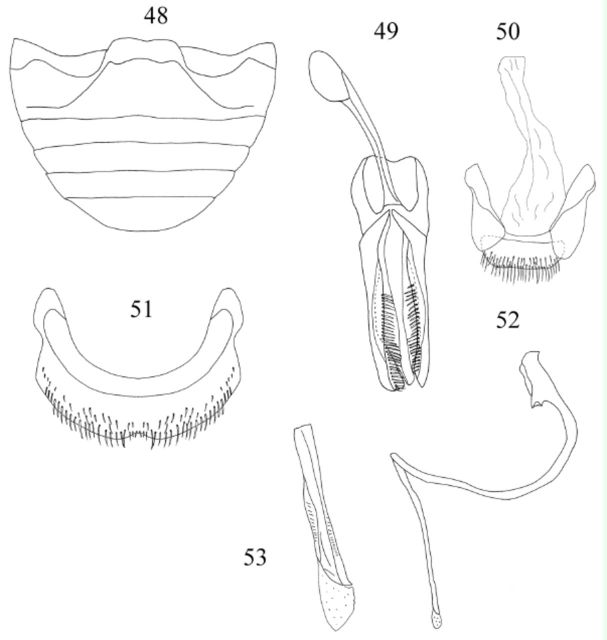
*Endochilus epipleuralis* Mader. 48: abdomen, male, ventral; 49: tegmen, inner view; 50: male genital segment, ventral; 51: abdominal segment VIII, male, ventral; 52: penis; 53: apex of penis. High quality figures are available online.

Abdomen ( Figures 48 , 135 ) with 5 ventrites in male; postcoxal lines of first ventrite joined medially; ventrite 1 along midline about 2.5 times longer than ventrite 2; ventrite 5 with posterior margin rounded. Abdominal segment VIII ( Figure 51 ) with posterior margin of sternite deeply emarginate medially. Male genital segment ( Figure 50 ); apophysis not observed.

Male genitalia as in Figures 49 , 52 , 53 . Penis guide as long as parameres; apex of penis ( Figure 53 ) short and beak-shaped.

Female unknown.

### Material examined

**Type material.** *Holotype* (male): “HOLOTYPUS/COLL, MUS. CONGO, Ma- yumbe: Gigo, 16-IV.1926, A. Collart/Holotypus, Epipleuralis/R. Det. F. 5838/Endochilus epipleuralis det. Mader” (RMCA).

**Distribution** . Democratic Republic of the Congo.

*Endochilus minor* Weise

( Figures 6 , 54–62 , 136–140 )

*Endochilus minor* Weise, 1898 : 121.

**Diagnosis** . This species is similar to *E. rubicundus* , but can be distinguished by its very broad and concave prosternal process, weakly explanate elytral margins and dark red pronotum. Penis with two acute apical projections of different length placed on inner and outer margin distinguishes this species from its congeners.

**Description** . Length 3.6–3.9; TL/EW = 0.97– 1.02; PL/PW = 0.27–0.30; EL/EW = 0.79– 0.81; PSL/PPW = 0.50.

Body ( Figure 6 ) with pronotal margins moderately broad; elytral margins weakly explanate. Head and pronotum dark red; labrum, ventral mouthparts, and antennae brownish. Scutellum dark red. Elytra predominantly bright red except dark red margins. Punctures on pronotum 2–3 diameters apart; punctures on elytra as on pronotum but shallower; dorsum glabrous except pronotal angles and elytral margins which are covered with sparse short setae. Ventral surface brown.

**Figures 136-144.**
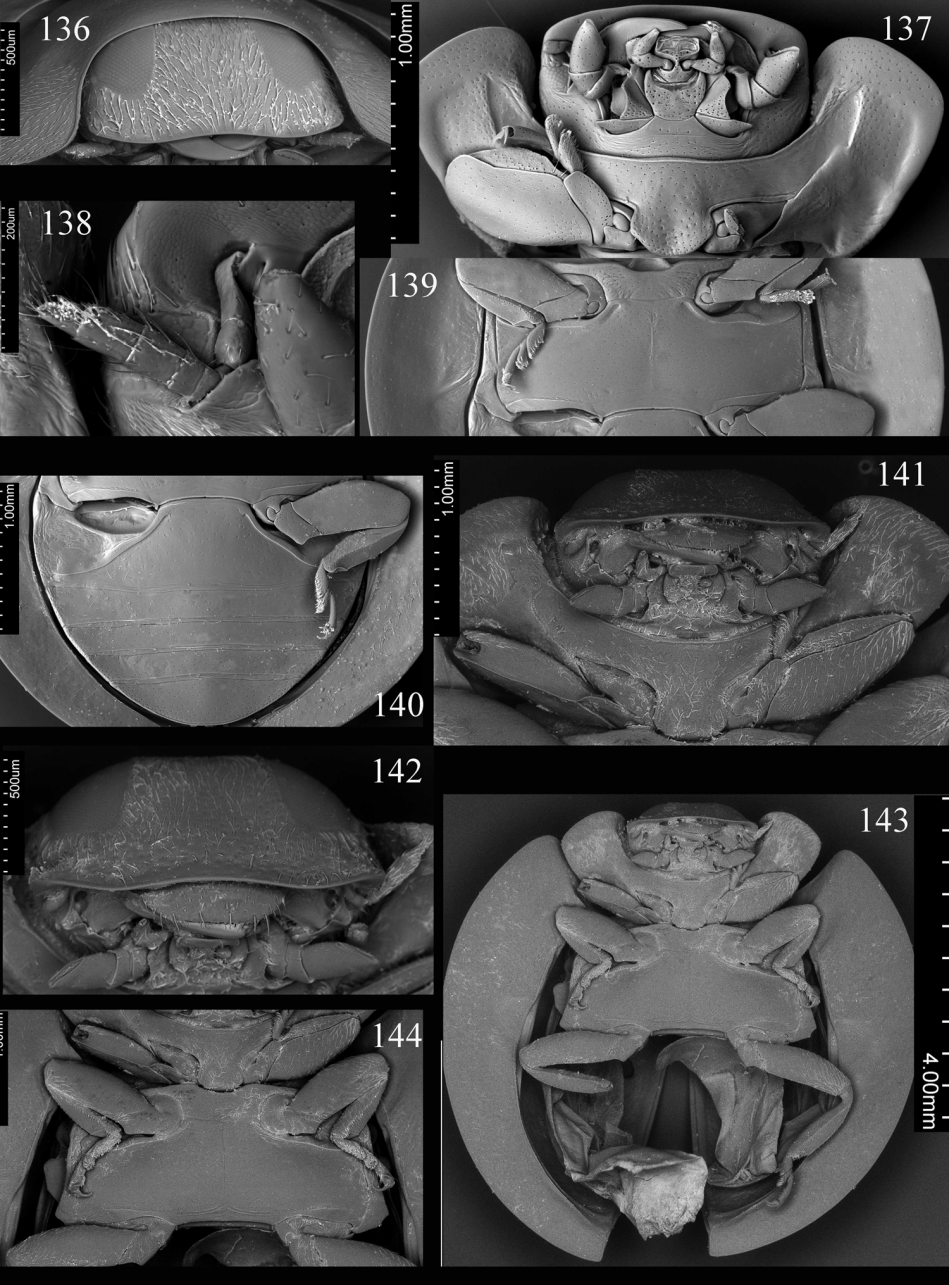
*Endochilus minor* Weise. 136: head, anterodorsal; 137: head, prothorax, ventral; 138: antenna; 139: meso- and metathorax, ventral; 140: abdomen, female, ventral. *E. niger* Fürsch. 141: head, prothorax, ventral; 142: head, anteroventral; 143: body, ventral view; 144: pro-, meso- and metathorax, ventral. High quality figures are available online.

Head flat medially, punctate, covered with rather dense and moderately long setae. Clypeus ( Figure 136 ) length anterior to eyes about 0.2 times head width, with almost straight anterior margin. Eyes large; interocular distance nearly 0.42 times head width; medial margins of eyes slightly rounded, divergent anteriorly. Maxillary terminal palpomere ( Figure 137 ) about 1.4 times longer than wide, lateral margin about 2 times as long as medial, subparallel along basal 1/2 of its length, moderately tapering apically. Antenna ( Figure 138 ) with penultimate antennomere about 1.42 times as long as terminal segment.

Prothorax about 0.88 times basal width of elytra; pronotal hypomeron with distinct fovea; prosternum smooth; prosternal process ( Figure 137 ) rounded at apex, distinctly punctate. Mesoventral process ( Figure 139 ) about 1.5 times mesocoxal longitudinal diameter, distinctly punctate. Elytral epipleuron ( Figure 139 ) about 10 times metanepisternum width, with distinct foveae.

Abdomen ( Figures 54 , 140 ) with 5 ventrites in both sexes; intercoxal process distinctly punctate, punctures 2–3 diameters apart; ventrite 1 along midline about 2.8 times longer than ventrite 2; ventrite 5 with posterior margin weakly emarginate in male, rounded in female. Abdominal segment VIII ( Figures 55 , 56 ) with posterior margin of sternite emarginate medially in male, rounded in female. Male genital segment ( Figure 58 ) with apophysis not observed.

Male genitalia as in Figures 57 , 61 , 62 . Penis guide slightly shorter than parameres; apex of penis ( Figure 62 ) with two acute projections of different length, longer placed on outer margin, shorter on inner margin apically.

Female genitalia as in Figures 59 , 60 . Sperm duct short, of two different diameters; spermatheca with spermathecal gland subcircular ( Figure 60 ).

### Material examined

**Type material.** *Lectotype (here designated)* (male): “Kamerun/Endochilus minor/ SYNTYPUS, Endochilus minor Weise, 1898 , labeled by MNHUB 2010” (MNB). ****Paralectotype**** : female: “25.4.96/N. Kamerun, Johann- Albrechtshőhe, L. Conradt S. 28, 72501/Endochilus minor m./SYNTYPUS, Endochilus minor Weise, 1898 , labeled by MNHUB 2010” (1: MNB).

**Other material:** “Kameroon/Endochilus rubicundus” (1: MNB); “Jaunde, 10.14, Endochilus rubicundus Weise, det. H.Fursch, 72” (1: MNB); “N.W. Kamerun, Moliwe b. Victoria, 17.I. – 7.III – 08, Frfr.v.Maltzan G.” (1: MNB).

**Distribution.** Cameroon.

*Endochilus niger* Fürsch

( Figures 7 , 63–69 , 141–144 )

*Endochilus niger* Fürsch, 1963 : 304.

**Diagnosis** . This species is similar to *E. styx* in having black dorsum, but is easily distinguished by its larger size and not arcuate anterior margin of clypeus. Apex of penis with long and slender apical process bent internally distinguishes this species from all its congeners.

**Description** . Length 4.8–5.0 mm; TL/EW = 0.98–1.00; PL/PW = 0.29–0.31; EL/EW = 0.80–0.86; PSL/PPW = 0.52.

Body ( Figure 7 ) with pronotal margins broad; elytral margins moderately explanate. Head and pronotum black; labrum, ventral mouthparts, and antennae dark reddish. Scutellum black. Elytra black. Punctures on pronotum 2.0–2.5 diameters apart; punctures on elytra 2–3 diameters apart; dorsum glabrous except pronotal angles and elytral margins which are covered with setae. Ventral surface dark red to dark brown.

rather dense and moderately long setae. Clypeus ( Figure 142 ) length anterior to eyes about 0.17 times head width, with almost straight anterior margin, weakly reflexed along anterior margin. Eyes large; interocular distance nearly 0.5 times head width; medial margins of eyes slightly rounded, divergent anteriorly. Maxillary terminal palpomere ( Figures 141 , 142 ) about 1.8 times longer than wide, lateral margin about 1.65 times as long as medial, subparallel along basal about 2/3 of its length, moderately tapering apically. Antenna with penultimate antennomere about 1.5 times as long as terminal antennomere.

Prothorax about 0.9 times basal width of elytra; pronotal hypomeron with shallow fovea; prosternum smooth; prosternal process ( Figure 141 ) covered with sparse long setae. Mesoventral process ( Figure 144 ) about 1.36 times mesocoxal longitudinal diameter, covered with sparse long setae. Elytral epipleuron ( Figure 143 ) about 9.5 times metanepisternum width, with distinct foveae.

**Figures 63-69.**
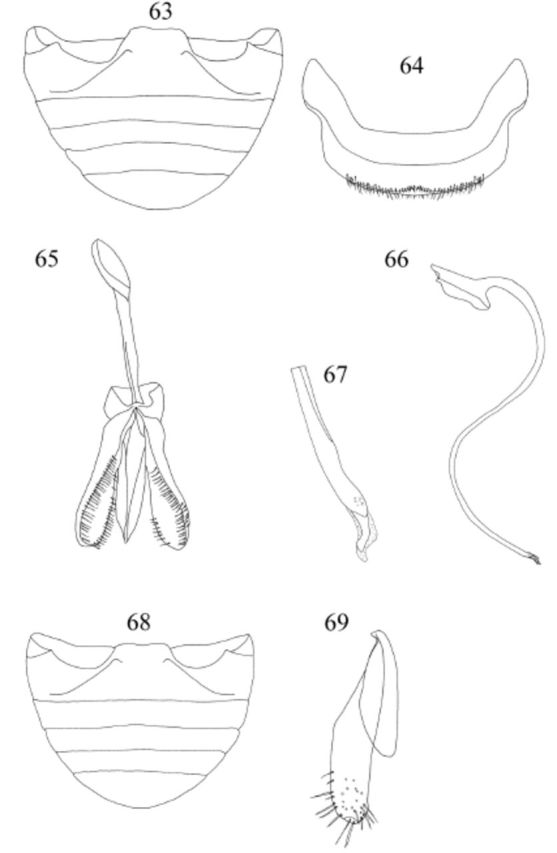
*Endochilus niger* Fürsch. 63: abdomen, male, ventral; 64: abdominal segment VIII, male; 65: tegmen, inner view; 66: penis; 67: apex of penis; 68: abdomen, female, ventral; 69: coxite, ventral. High quality figures are available online.

Abdomen ( Figures 63 , 68 ) with 5 ventrites in both sexes; ventrite 1 along midline about 2.5 times longer than ventrite 2; ventrite 5 with posterior margin subtruncate in male and rounded in female. Abdominal segment VIII ( Figure 64 ) with posterior margin of sternite emarginate medially.

Male genitalia as in Figures 65 , 66 , 67 . Penis guide as long as parameres; apex of penis ( Figure 67 ) with long and slender apical process bent internally.

Female genitalia as in Figure 69 . Studied only part of ovipositor.

### Material examined

**Type material.** *Holotype* (male): “3 Peu. 57/NIMBA (Guinea), Lamotte, Amiet, Vanderplaetsen, XII.56 – V- 57/Holotypus, Endochilus niger Fürsch” (MNHN). ****Paratype**** female: “NIMBA (Guinea), Lamotte, Amiet, Vanderplaetsen, XII.56 – V- 57/5 mars 57/Allotypus, Endochilus niger Fürsch” (1: MNHN).

**Distribution.** Guinea

*Endochilus plagiatus* Sicard

( Figures 8 , 70–78 , 145–149 )

*Endochilus plagiatus* Sicard, 1920 : 211.

**Diagnosis** . Black patch along basal half length of elytral suture with combination of dark red coloration and pubescence covered borders of elytral discs distinguish this species from all its congeners.

**Description** . Length 3.15–3.18 mm; TL/EW = 1.02–1.05; PL/PW = 0.18–0.20; EL/EW = 0.85–0.87; PSL/PPW = 0.52.

**Figures 145-154.**
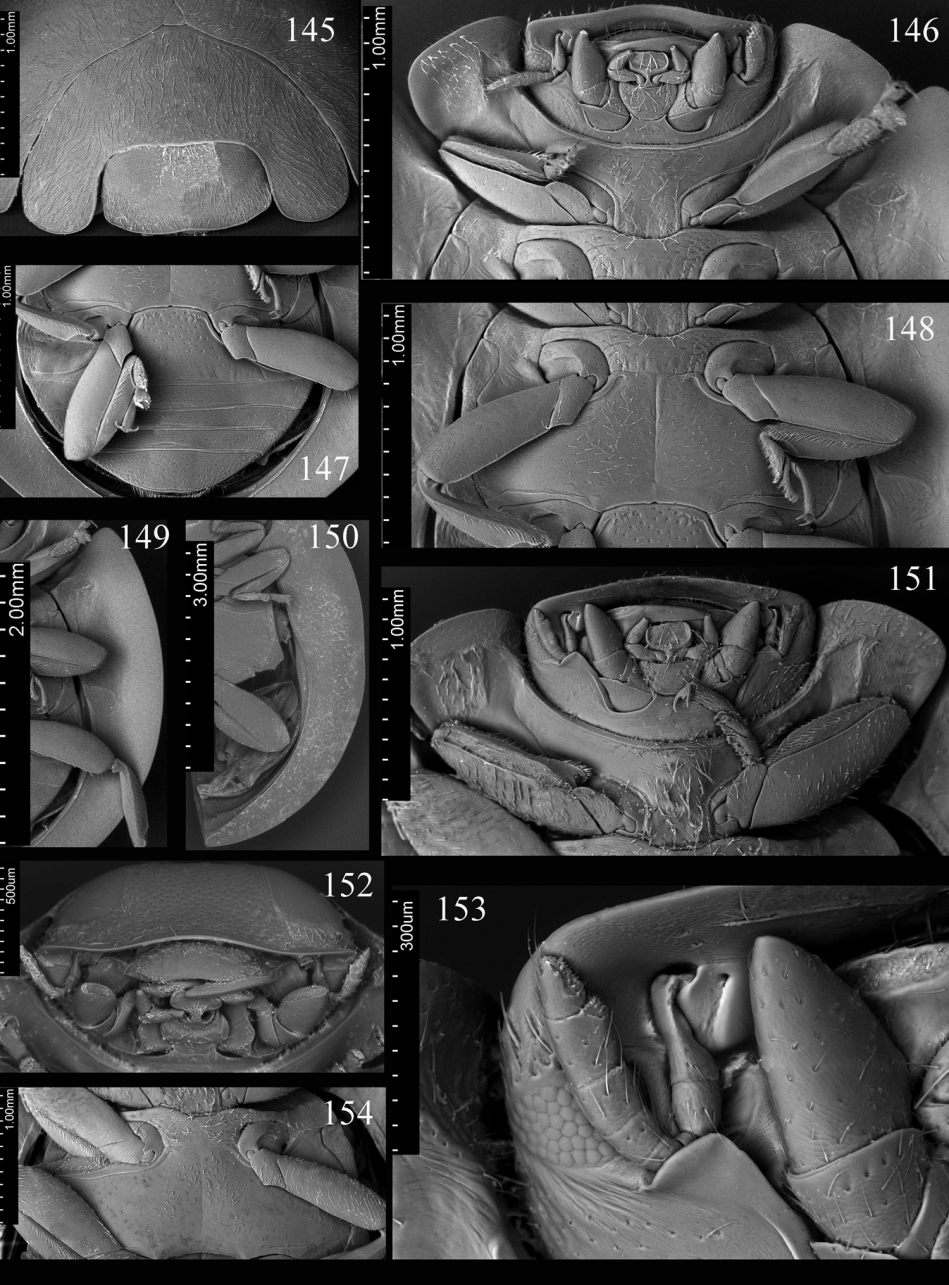
*Endochilus plagiatus* Sicard. 145: head, prothorax and base of elytra, anterodorsal; 146: head, prothorax, ventral; 147: abdomen, male, ventral; 148: meso- and metathorax, ventral; 149: elytral epipleuron. *E. rubicundus* Weise. 150: elytral epipleuron; 151: head, prothorax, ventral; 152: head, anteroventral; 153: antenna, maxillary palpus, ventral; 154: meso- and metathorax, ventral. High quality figures are available online.

Body ( Figure 8 ) with pronotal margins moderately broad, weakly visible from above; elytral margins moderately explanate. Head and pronotum dark red; labrum, ventral mouthparts, and antennae brownish. Scutellum black. Elytra predominantly dark red with black patch running from base to mid length of elytral suture, black bordering around elytral discs and bright red margins. Punctures on pronotum 1.5–2.0 diameters apart; punctures on elytra slightly sparser and shallower than those on pronotum, 2–3 diameters apart; dorsum glabrous except pronotum, elytral base, and margins, which are covered with setae. Ventral surface dark red to brownish.

dense and long setae. Clypeus ( Figure 145 ) length anterior to eyes about 0.22 times head width, weakly arcuate anteriorly, weakly reflexed along anterior margin. Eyes large; interocular distance nearly 0.41 times head width; medial margins of eyes slightly rounded, divergent anteriorly. Maxillary terminal palpomere ( Figure 146 ) about 1.7 times longer than wide, lateral margin about 2 times as long as medial, subparallel along basal 1/2 of its length, moderately tapering apically. Antenna as in Figure 146 with penultimate antennomere about 1.42 times as long as terminal segment.

Prothorax about 0.9 times basal width of elytra; pronotal base bordered; pronotal hypomeron with distinct fovea; prosternum smooth; prosternal process ( Figures 146 , 148 ). Mesoventral process ( Figure 148 ) about 1.38 times mesocoxal longitudinal diameter. Elytral epipleuron ( Figure 149 ) about 9 times metanepisternum width, with distinct foveae.

**Figures 70-78.**
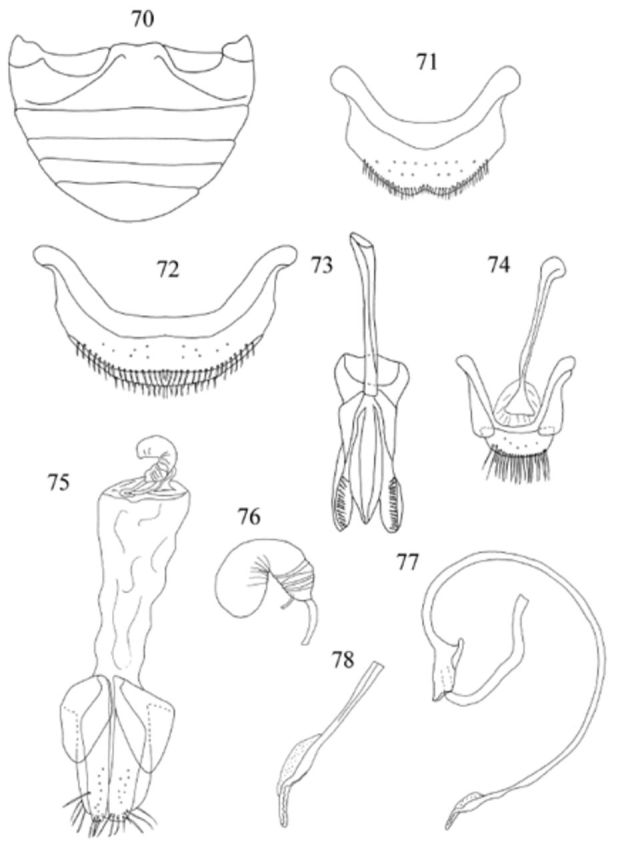
*Endochilus plagiatus* Sicard. 70: abdomen, female, ventral; 71: abdominal segment VIII, male, ventral; 72: abdominal segment VIII, female, ventral; 73: tegmen, inner view; 74: male genital segment, ventral; 75: female genitalia; 76: spermatheca; 77: penis; 78: apex of penis. High quality figures are available online.

Abdomen ( Figures 70 , 147 ) with 5 ventrites in both sexes; intercoxal process distinctly punctate 2–3 diameters apart; ventrite 1 along midline about 2.7 times longer than ventrite 2; ventrite 5 with posterior margin truncate in male, rounded in female. Abdominal segment VIII ( Figures 71 , 72 ) with posterior margin of sternite emarginate medially in male, truncate in female. Male genital segment ( Figure 74 ) with apophysis at base distinctly swollen.

Male genitalia as in Figures 73 , 77 , 78 . Penis guide slightly shorter than parameres; apex of penis ( Figure 78 ) with long serrate process. Female genitalia as in Figures 75 , 76 . Spermathecal gland not observed.

### Material examined

**Type material.** *Lectotype (here designated)* (male): “San Thome/ Endochilus plagiatus Sic., Type/Syntype” (MNHN). ****Paralectotypes**** : “San Thome/ Endochilus plagiatus Sic., Type/Syntype” (4: MNHN); “St. Thome, Syntype” (4: MNHN).

**Other material** : “Sao Tome, on coconut, iv. 1956, F.J. Simmonds, C I.E.. Coll. 14731” (1: NHM); “Port W. Africa, Sao Tome, taken from coffee plants. 1956” (2: NHM); “Endochilus plagiatus” (1: MNHN).

**Distribution.** São Tomé and Principe.

*Endochilus rubicundus* Weise

( Figures 9 , 79–87 , 150–154 )

*Endochilus rubicundus* Weise, 1898 : 120.

**Diagnosis** . This species is most similar to *E. minor* , but can be distinguished by narrower and flat prosternal process; moderately explanate elytral margins, and brighter coloration of pronotum. Apex of penis rounded and simple distinguishes this species from all its congeners.

**Description** . Length 5.14–5.16 mm; TL/EW = 0.98–1.02; PL/PW = 0.28–0.30; EL/EW = 0.85–0.87; PSL/PPW = 0,56.

Body ( Figure 9 ) with pronotal margins moderately broad; elytral margins moderately explanate. Head dark red; pronotum bright red; labrum, ventral mouthparts and antennae brownish. Scutellum dark red. Elytra predominantly bright red except dark red margins. Punctures on pronotum 1.5–2.5 diameters apart; punctures on elytra as on pronotum but shallower; dorsum glabrous except pronotal angles and elytral margins which are covered with sparse short setae. Ventral surface brownish.

Head flat medially, punctate, covered with sparse short setae. Clypeus ( Figure 152 ) length anterior to eyes about 0.17 times head width, with almost straight anterior margin. Eyes large; interocular distance nearly 0.56 times head width; medial margins of eyes slightly rounded, almost parallel. Maxillary terminal palpomere ( Figures 151 , 153 ) about 1.4 times longer than wide, lateral margin about 2.7 times as long as medial, subparallel along basal 1/3 of its length, moderately tapering apically. Antenna ( Figure 153 ) with penultimate antennomere about 1.15 times as long as terminal segment.

Prothorax about 0.9 times basal width of elytra; pronotal hypomeron with distinct fovea; prosternum smooth; prosternal process ( Figure 151 ) covered with sparse long setae. Mesoventral process ( Figure 154 ) about 1.35 times mesocoxal longitudinal diameter, covered with sparse long setae. Elytral epipleuron ( Figure 150 ) about 9.5 times metanepisternum width, with distinct foveae.

**Figures 79-87.**
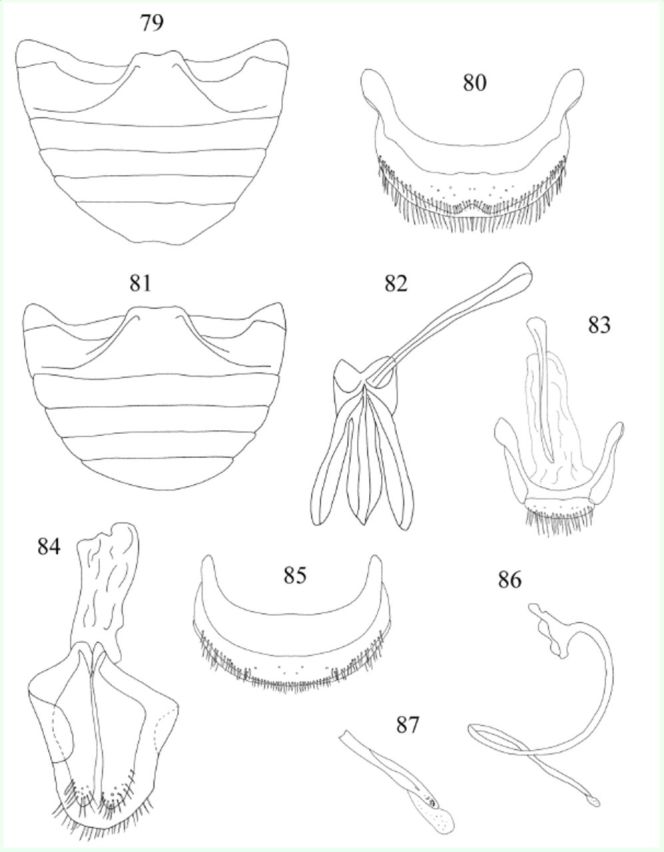
*Endochilus rubicundus* Weise. 79: abdomen, male, ventral; 80: abdominal segment VIII, male, ventral; 81: abdomen, female, ventral; 82: tegmen, inner view; 83: male genital segment, ventral; 84: female genitalia; 85: abdominal segment VIII, female, ventral; 86: penis; 87: apex of penis. High quality figures are available online.

Abdomen ( Figures 79 , 81 ) with 5 ventrites in both sexes; ventrite 1 along midline about 2.6 times longer than ventrite 2; ventrite 5 with posterior margin weakly emarginate in male, rounded in female. Abdominal segment VIII ( Figures 80 , 85 ) with posterior margin of sternite emarginate medially in male, rounded in female. Male genital segment ( Figure 83 ) with long apophysis, narrow, simple at base and at apex.

Male genitalia as in Figures 82 , 86 , 87 . Penis guide slightly shorter than parameres; apex of penis ( Figure 87 ) simple, rounded.

Female genitalia as in Figure 84 . Spermatheca not studied.

### Material examined

**Type material.** *Lectotype (here designated)* **(** male): “S.O. Kamerun, Lolodorf, 19.II- 7.VI.95, L. Conradt S./72499/32/Endochilus rubicundus/SYNTYPUS, Endochilus rubicundus Weise, 1898 , labeled by MNHUB 2010.” (MNB). ****Paralectotype**** , female: “Kamerun Kraah/Endochilus rubicundus/ labeled by MNHUB 2010.” (1: MNB)

**Distribution.** Cameroon.

*Endochilus styx* Sicard

( Figures 10 , 88–96 , 155–159 )

*Endochilus styx* Sicard, 1911 : 289.

**Diagnosis** . This species is similar to *E. niger* by the colouration, but is distinguished by smaller size and weakly arcuate middle part of anterior margin of clypeus. Apex of penis subacute and weakly bent internally distinguishes this species from all its congeners **.**

**Description** . Length 2.89–3.00 mm; TL/EW = 1.00–1.03; PL/PW = 0.28–0.30; EL/EW = 0.80–0.82; PSL/PPW = 0.54.

Body ( Figure 10 ) with pronotal margins moderately broad; elytral margins moderately explanate. Head and pronotum black; labrum, ventral mouthparts, and antennae dark brownish to blackish. Scutellum black. Elytra black. Punctures on pronotum 2–3 diameters apart; punctures on elytra as on pronotum but shallower; dorsum glabrous except pronotal angles and elytral margins, which are covered with setae. Ventral surface black except brownish abdomen.

**Figures 155-164.**
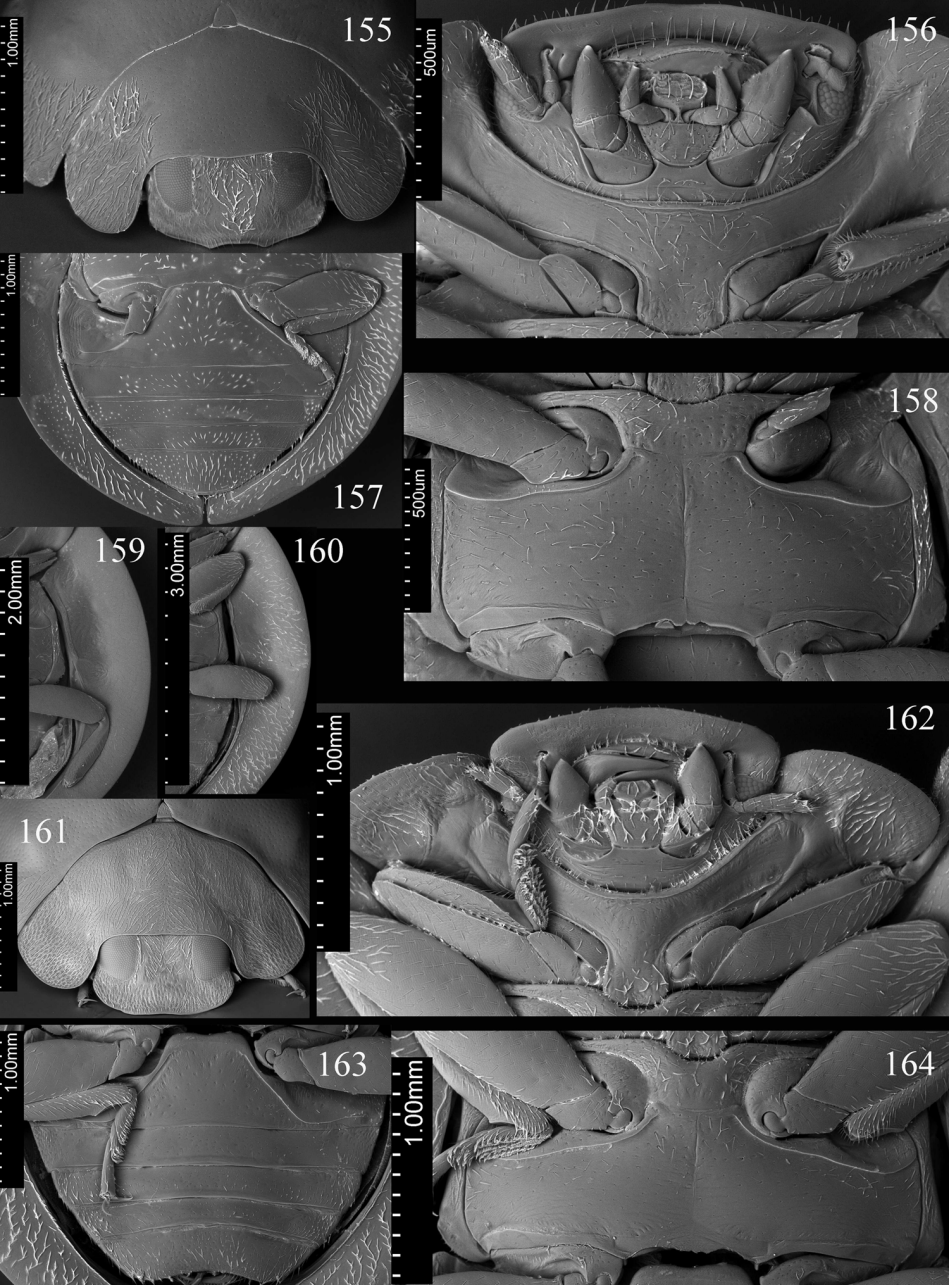
*Endochilus styx* Sicard. 155: head, prothorax and base of elytra, anterodorsal; 156: head, prothorax, ventral; 157: abdomen, male, ventral; 158: meso- and metathorax, ventral; 159: elytral epipleuron. *E. weisei* Mader. 160: elytral epipleuron; 161: head, prothorax and base of elytra, anterodorsal; 162: head, prothorax, ventral; 163: abdomen, male, ventral; 164: meso- and metathorax, ventral. High quality figures are available online.

Head flat medially, punctate, covered with sparse short setae. Clypeus ( Figure 155 ) length anterior to eyes about 0.15 times head width, with arcuate middle part of anterior margin. Eyes moderately large; interocular distance nearly 0.46 times head width; medial margins of eyes almost parallel. Maxillary terminal palpomere ( Figure 156 ) about 1.42 times longer than wide, lateral margin about 2.5 times as long as medial, subparallel along basal 1/3 of its length, moderately tapering apically. Antenna as in Figure 156 with penultimate antennomere about 1.15 times as long as terminal segment.

Prothorax about 0.85 times basal width of elytra; pronotal hypomeron with shallow fovea; prosternum smooth; prosternal process ( Figure 156 ) covered with sparse long setae.

Mesoventral process ( Figure 158 ) about 1.4 times mesocoxal longitudinal diameter, covered with sparse long setae. Elytral epipleuron ( Figure 159 ) about 11 times metanepisternum width, with distinct foveae.

**Figures 88-96.**
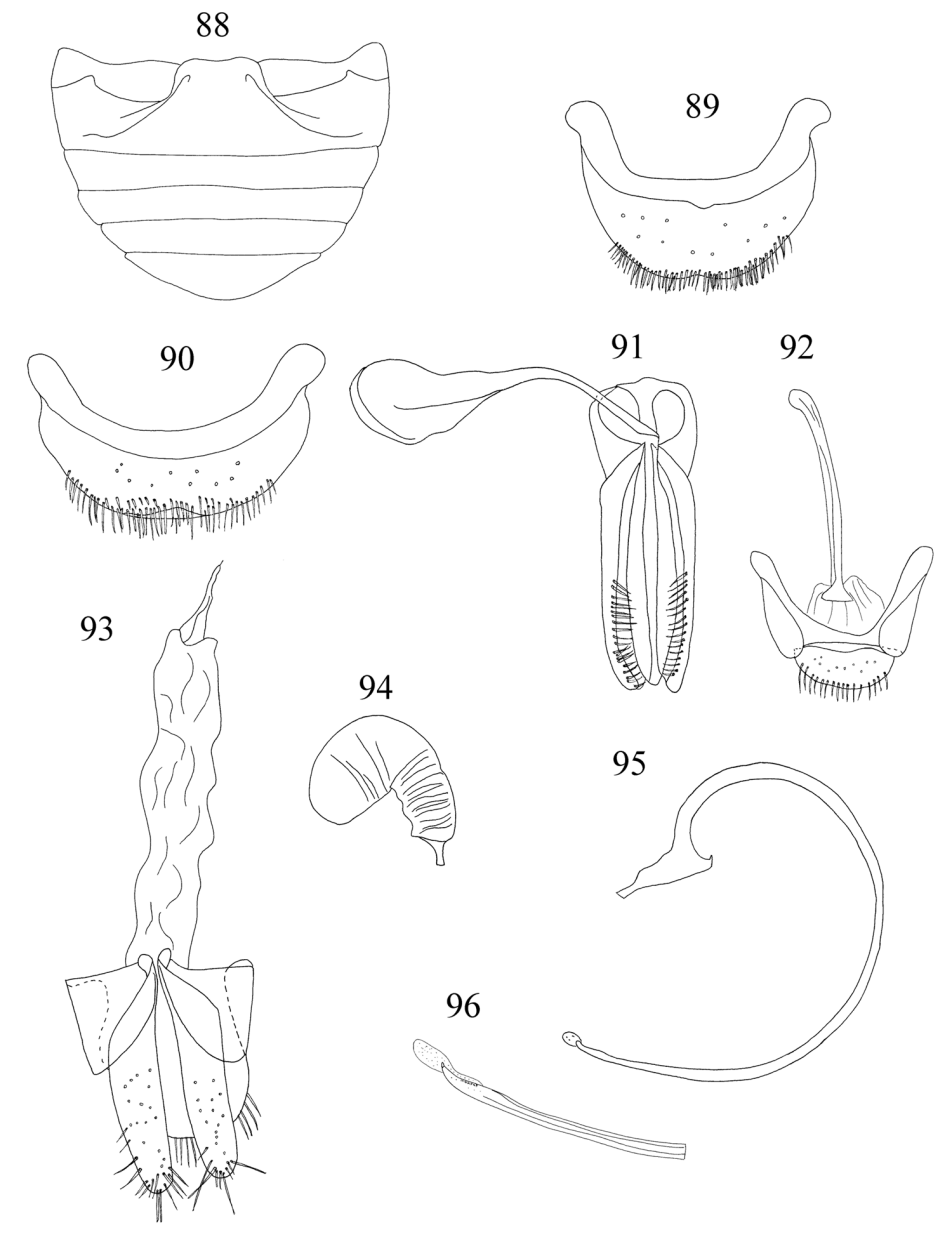
*Endochilus styx* Sicard. 88: abdomen, female, ventral; 89: abdominal segment VIII, male, ventral; 90: abdominal segment VIII, female, ventral; 91: tegmen, inner view; 92: male genital segment, ventral; 93: female genitalia; 94: spermatheca; 95: penis; 96: apex of penis. High quality figures are available online.

Abdomen ( Figures 88 , 157 ) with 5 ventrites in both sexes; ventrite 1 along midline about 2.7 times longer than ventrite 2; ventrite 5 with posterior margin rounded in both sexes. Abdominal segment VIII with posterior margin of sternite weakly emarginate medially in male and female ( Figures 89 , 90 ). Male genital segment ( Figure 92 ) with apophysis at base distinctly swollen.

Male genitalia as in Figures 91 , 95 , 96 . Penis guide as long as parameres; apex of penis ( Figure 96 ) subacute and weakly bent internally.

Female genitalia as in Figures 93 , 94 . Spermathecal gland absent.

### Material examined

**Type material.** *Lectotype (here designated)* (male): “Is. Principe, Bahia do Oeste, V- VI.1901, 100 – 200m, L. Fea./Syntype” (MNHN). ****Paralectotypes**** : “Is. Principe, Bahia do Oeste, V-VI.1901, 100 – 200m, L. Fea./Syntype” (1: MNHN); “Endochilus styx Sic., Typus!, Is. Principe, Bahia do Oeste, V- VI.1901, 100 – 200m, L. Fea./Syntype” (1: MNHN).

**Other material** : “PRINCIPE, Esperanza, iv, 1956, F.J. Simmonds, C.I.E. Coll. 14731, Press by Com. Inst. Ent., B.M. 1956-30” (3: NHM); Principe, 1956, F.J. Simmonds, coconut” (1: NHM); “PRINCIPE, Esperanza, iv, 1956, F.J. Simmonds, C.I.E. Coll. 14731, Endochilus styx Sic., R.D. Pope det., from deser. 1956, Press by Com. Inst. Ent., B.M. 1956- 30” (1: NHM).

**Distribution.** São Tomé and Principe.

*Endochilus weisei* Mader

( Figures 11 , 97–105 , 160–164 )

*Endochilus weisei* Mader, 1954 : 70.

**Diagnosis** . This species resembles *E. compater* and *E. cavifrons* but can be distinguished by smaller size and bright brown elytra with dark red margins. Penis with row of teeth placed on outer and inner margin before apex and stout bottle-opener-shaped apex distinguish this species from all its congeners.

**Description** . Length 4.1–4.2 mm; TL/EW = 1.01–1.03; PL/PW = 0.25–0.28; EL/EW = 0.80–0.85; PSL/PPW = 0.62.

Body ( Figure 11 ) with pronotal margins very broad; elytral margins widely explanate. Head and pronotum black; labrum, ventral mouthparts, and antennae brownish. Scutellum black. Elytra predominantly bright brown except margins, which are black. Punctures on pronotum 1.5–2.0 diameters apart; punctures on elytra slightly sparser and shallower than those on pronotum, 2–3 diameters apart; dorsum glabrous except pronotum and elytral margins, which are covered with setae. Ventral surface dark reddish to brown.

Head flat medially, punctate, covered with dense and long setae. Clypeus ( Figure 161 ) length anterior to eyes about 0.21 times head width, weakly arcuate anteriorly. Eyes large; interocular distance nearly 0.43 times head width; medial margins of eyes slightly rounded, almost parallel. Maxillary terminal palpomere ( Figure 162 ) about 1.42 times longer than wide, lateral margin about 2.6 times as long as medial, subparallel along basal 1/3 of its length, moderately tapering apically. Antenna as in Figure 162 with penultimate antennomere about 2 times as long as terminal segment.

Prothorax about 0.9 times basal width of elytra; pronotal hypomeron with distinct fovea; prosternum smooth; prosternal process ( Figure 162 ). Mesoventral process ( Figure 164 ) about 0.94 times mesocoxal longitudinal diameter. Elytral epipleuron ( Figure 160 ) about 11 times metanepisternum width, with shallow foveae.

**Figures 97-105.**
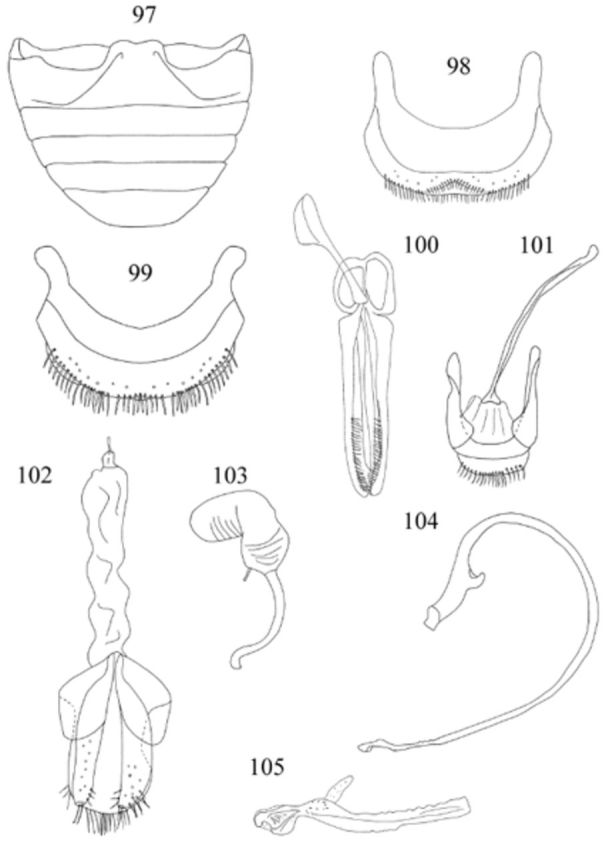
*Endochilus weisei* Mader. 97: abdomen, female, ventral; 98: abdominal segment VIII, male, ventral; 99: abdominal segment VIII, female, ventral; 100: tegmen, inner view; 101: male genital segment, ventral; 102: female genitalia; 103: spermatheca; 104: penis; 105: apex of penis. High quality figures are available online.

Abdomen ( Figures 97 , 163 ) with 5 ventrites in both sexes; intercoxal process distinctly punctate, 2–3 diameters apart; ventrite 1 along midline about 2 times longer than ventrite 2; ventrite 5 with posterior margin strongly emarginate in male, rounded in female. Abdominal segment VIII with posterior margin of sternite emarginate medially in male, weakly in female ( Figures 98 , 99 ). Male genital segment ( Figure 101 ) with apophysis at base distinctly swollen.

Male genitalia as in Figures 100 , 104 , 105 . Penis guide as long as parameres; penis with row of teeth placed on outer and inner margin before apex; apex of penis ( Figure 105 ) stout bottle-opener-shaped.

Female genitalia as in Figures 102 , 103 . Spermathecal gland not observed.

### Material examined

**Type material.** *Holotype* (male): “Holotypus/Congo-Belge: P.N.A., Mutsora 1939, Hackars/Coll. Mus. Congo, (ex. coll. I.P.N.C.B.)/Holotypus Weisei m/ *Endochilus* Weise, n.sp., L. Mader det, 1952” (RMCA).

**Other material** : “Kamerun Barombi Conradt/Endochilus spec., det. Korschefsky 1939/Mus. Zool. Polonicum, Warszawa, 12/45” (3: MIZ); “Kamerun, Joh- Albrechtshőhe, 3.-28.VIII.98, L. Conradt S./ *Endochilus weisei* Mader, det. H.Fürsch 73” (2: MNB).

**Distribution** . Cameroon, Democratic Republic of the Congo.

**Key to the species of** *Endochilus*

1. Dorsum entirely black ( Figures 7 , 10 )…..10 - Dorsum mostly bright, dark red or bright brown………………………………………..2

2. Elytra with black patch along basal half lenght of suture ( Figure 8 )................................ …………………………… *E. plagiatus* Sicard - Elytra without patch along suture...............3

3. Postcoxal lines of first ventrite joined medially ( Figures 48 , 135 )……………….……… ………………………... *E. epipleuralis* Mader - Postcoxal lines of first ventrite separate medially ( Figures 54 , 140 )…….……….……4

4. Distance between postcoxal lines of first ventrite equal 0.30 width of intercoxal process ( Figures 12 , 108 ) [elytra very intense dark red ( Figure 1 )]……...…... *E. abdominalis***sp. nov.** - Postcoxal lines of first ventrite widely separated medially……………………………….5

5. Elytral margins widely explanate covered with dense, long setae ( Figures 3 , 4 , 11 ); pronotum black covered with dense, long setae; posterior margin of ventrite 5 strongly emarginate in male………….………………...…..6 - Elytral margins weakly or moderately explanate covered with sparse, short setae ( Figures 2 , 6 , 9 ); pronotum bright-, dark red or blackish with only pronotal angles covered with sparse, short setae; posterior margin of ventrite 5 weakly emarginate in male…….....8

6. Elytra bright brown; elytral margins dark red ( Figure 11 ); abdomen with 5 ventrites in both sexes; body less than 4.2 mm.......................................... *E. weisei* Mader - Elytra bright red; elytral margins black ( Figures 3 , 4 ); abdomen with 6 ventrites in male, 5 in female; body above 5.5 mm……...7

7. Base of elytra bright red ( Figure 4 ); posterior margin of ventrite 5 in female rounded ( Figure 41 ) ………...…….. *E. compater* Weise - Base of elytra black ( Figure 3 ); posterior margin of ventrite 5 in female weakly emarginat.................................... *E. cavifrons* Weise

8. Elytral margins moderately explanate ( Figure 9 ); pronotum bright red; apex of penis simple ( Figure 87 ).......... *E. rubicundus* Weise - Elytral margins weakly explanate ( Figures 2 , 6 ); pronotum blackish or dark red; apex of penis with one or two acute projections.........9

9. Pronotum blackish; elytra dark red ( Figure 2 ); apex of penis with acute projection placed outwardly ( Figure 29 )....................................... …………………….. *E. brunneocinctus* Sicard - Pronotum dark red; elytra bright red ( Figure 6 ); apex of penis with two acute projections of different length placed on inner and outer margin ( Figure 62 )............................ ………………………………. *E. minor* Weise

10. Clypeus with arcuate anterior margin ( Figure 155 ); body less than 3 mm; apex of penis subacute and weakly bent internally…………………………...….. *E. styx* Sicard - Clypeus with not arcuate anterior margin ( Figure 142 ); body longer than 4.8 mm; apex of penis with long and slender apical process bent internally…………..…... *.E. niger* Fürsch

## Discussion

*Endochilus* is a homogenous and not speciose group. There are few differences between species of this genus, based mainly on shapes of penis apex, sizes of body, shades of coloration, arrangement of postcoxal lines on abdominal process, or presence of foveolation on hypomera and epipelura.

*Endochilus* shares various characters with other five Afrotropical genera of Chilocorini. It shares 8-segmented antennae with *Brumoides* Chapin; arrangement of metaventral and abdominal postcoxal lines resemble those in *Chilocorus* Leach; moderately stout tibia and simple tarsal claws are similar to *Brumus* Mulsant and *Brumoides* ; spermatheca simple without membranous beak resembles that in *Exochomus* Redten- bacher. In particular *Endochilus* shares numerous features with *Chapinaria* Łączyński *et* Tomaszewska, such as characters of maxil- lae, labium, foveolate hypomera and elytral epipleura, shape of meso-metaventrite junction, arrangement of metaventral postcoxal lines, and pattern of dorsal coloration.

*Endochilus* , however, differs from all other Chilocorini genera in having labrum entirely hidden under clypeus, lateral margins of pronotum and elytra setose, antennal grooves on a head deep but short, clypeus long in front of eyes and expanded laterally into eyes almost dividing each eye in two parts, prosternal and mesoventral processes very broad, elytral epipleura very broad, and sperm duct short and only slightly longer or as long as spermatheca.

The phylogenetic relationships between Chilocorini genera are, however, still unclear and hopefully will be resolved by the thorough cladistic analysis of the entire tribe.
